# Optimal COVID-19 quarantine and testing strategies

**DOI:** 10.1038/s41467-020-20742-8

**Published:** 2021-01-07

**Authors:** Chad R. Wells, Jeffrey P. Townsend, Abhishek Pandey, Seyed M. Moghadas, Gary Krieger, Burton Singer, Robert H. McDonald, Meagan C. Fitzpatrick, Alison P. Galvani

**Affiliations:** 1 Center for Infectious Disease Modeling and Analysis (CIDMA), Yale School of Public Health, New Haven, CT 06520 USA; 2 Department of Biostatistics, Yale School of Public Health, New Haven, CT 06510 USA; 3 Department of Ecology and Evolutionary Biology, Yale University, New Haven, CT 06525 USA; 4 Program in Computational Biology and Bioinformatics, Yale University, New Haven, CT 06511 USA; 5 Program in Microbiology, Yale University, New Haven, CT 06511 USA; 6 Agent-Based Modelling Laboratory, York University, Toronto, ON M3J 1P3 Canada; 7 NewFields E&E, Boulder, CO 80301 USA; 8 Skaggs School of Pharmacy and Pharmaceutical Science, University of Colorado Anschutz Medical Campus, Aurora, CO 80045 USA; 9 Emerging Pathogens Institute, University of Florida, P.O. Box 100009, Gainesville, FL 32610 USA; 10 Group Health, Safety and Environment; BHP, Melbourne, VIC 3000 Australia; 11 Center for Vaccine Development and Global Health, University of Maryland School of Medicine, Baltimore, MA 21201 US

**Keywords:** Computational models, SARS-CoV-2, Health policy, Population screening

## Abstract

For COVID-19, it is vital to understand if quarantines shorter than 14 days can be equally effective with judiciously deployed testing. Here, we develop a mathematical model that quantifies the probability of post-quarantine transmission incorporating testing into travel quarantine, quarantine of traced contacts with an unknown time of infection, and quarantine of cases with a known time of exposure. We find that testing on exit (or entry and exit) can reduce the duration of a 14-day quarantine by 50%, while testing on entry shortens quarantine by at most one day. In a real-world test of our theory applied to offshore oil rig employees, 47 positives were obtained with testing on entry and exit to quarantine, of which 16 had tested negative at entry; preventing an expected nine offshore transmission events that each could have led to outbreaks. We show that appropriately timed testing can make shorter quarantines effective.

## Abstract (web summary)

Safely reducing the necessary duration of quarantine for COVID-19 could lessen the economic impacts of the pandemic. Here, the authors demonstrate that testing on exit from quarantine is more effective than testing on entry, and can enable quarantine to be reduced from fourteen to seven days.

## Introduction

The COVID-19 pandemic has engendered unprecedented efforts to quell ongoing outbreaks and manage healthcare capacity, including strict travel restrictions and stay-at-home orders. These efforts have disrupted workplaces, leading to significant and pervasive socioeconomic costs^1,2^. In turn, these economic pressures have led many governments and corporations to lift restrictions^3^. Safely reopening in the absence of a vaccine relies on reducing the likelihood of an infectious individual entering a workplace, school, or other social gathering^4^. Current strategies to ensure safety often include a 14-day quarantine—either as a consequence of travel or following exposure to an infected person, as recommended by the World Health Organization (WHO)^5^. These quarantines are sometimes combined with entry and/or exit testing, in which a positive test prompts isolation until recovery.

Quarantine imposes myriad challenges for institutions of government, militaries, businesses, universities, and other entities. At the individual level, the recommended 14-day quarantine causes strain on mental health^6,7^. This burden is coupled with the associated economic toll and potential impacts on operational integrity. For example, the typical 14-day on-and-off cycle for offshore oil and gas employees is substantially disrupted when quarantine measures are required. These quarantines result in prolonged time periods that crew members are away from their homes. Given the impact of long quarantines on mental health^6,7^, we evaluated the potential that a shorter quarantine combined with testing optimization could achieve reduced transmission of COVID-19 within close-quarter environments where there is a potentially high risk for rapid spread.

Evidence suggests that isolation of cases upon symptom onset is insufficient to contain an outbreak of COVID-19^8^. The probability of transmission can be reduced substantially through quarantine and testing^4^. Previous work has focused on the impact of quarantine and testing on population-level COVID-19 incidence and deaths^9–11^, shortened quarantines upon negative reverse-transcription polymerase chain reaction (RT-PCR) test at entry from contact tracing or 7 days after exposure^12^ and testing measures that are most appropriate for disease surveillance within a high-risk population (e.g., health-care workers) by examining various testing frequencies and their reduction of secondary infections^13^. Currently, there is no consensus regarding the optimal duration of quarantine or timing of testing that minimizes the probability of post-quarantine transmission (PQT), defined as one or more infections observed after the quarantine period. Many institutions are relying on testing at entry into quarantine combined with other measures such as symptom screenings, hand sanitizers, and face masks to reduce the risk of an outbreak. However, the majority of COVID-19 transmission is attributable to presymptomatic and asymptomatic cases, making screening for symptoms alone inadequate to prevent or interrupt a COVID-19 outbreak^8^. In addition, testing too early post-infection is likely to produce a false-negative result^14^. Thus, symptom-based screening and one-time testing could still entail a significant probability of PQT.

Some jurisdictions have suggested and implemented testing upon exit from a 14-day quarantine^15^. For example, Australia has implemented a compulsory 14-day quarantine, with testing within 48 h after arrival and between days 10 and 12 of quarantine, to reduce transmission from imported cases^16^. Although these multiple tests aid in case identification, this strategy does not include any reduction of the burden of long quarantine. Understanding the complementarity of quarantine and testing in reducing PQT would provide vital insight into effective strategies that mitigate disease spread in travel-based and contact-tracing based contexts. We applied a mathematical modeling approach to evaluate whether a less burdensome quarantine and testing strategy exist that would be epidemiologically equivalent to the standard 14-day quarantine protocol in reducing PQT. This model accounts for the infectivity profile of an infected individual as well as the temporal diagnostic sensitivity of RT-PCR testing. Across a variety of quarantine and testing scenarios, we estimated the probability of PQT for an infected individual who has not manifested symptoms by the end of the quarantine period. We considered three applications: (i) quarantine for travel, initiated at random times across the infectious course, (ii) quarantine prompted by contact-tracing and therefore initiated early in the infectious course, and (iii) quarantine when the time of exposure is known. We compared the probability of PQT under three testing scenarios: (i) on entry to quarantine only, (ii) on exit from quarantine only, and (iii) on both entry to and exit from quarantine for an infected individual. Across these scenarios, we varied the duration of quarantine and identified the optimal testing date based on that duration. As validation of our recommendations, we analyzed the real-world application of our model-based findings to protocols within the oil and gas industry that prevented offshore transmission.

## Results

We derived an infectivity profile based on transmission pairs of COVID-19 infected individuals^17^, a basic reproduction number of *R*_0_ = 2.5, and an incubation period of 8.29 days^18^, and estimated the temporal diagnostic sensitivity of RT-PCR tests^19^ (Supplementary Table 1). Specifying 30.8% of infections as remaining asymptomatic across the disease course^20,21^, we estimated that perfect isolation of cases upon symptom onset would reduce the reproduction number to 1.6, with 1.2 secondary cases occurring during the incubation period (Supplementary Fig. 1A). The reproductive number remained above one even when we lowered the asymptomatic proportion to 22.6% or reduced *R*_0_ to 2 (Supplementary Fig. 1B–D). Therefore, perfect isolation of all symptomatic individuals would not be sufficient to interrupt the chain of disease transmission.

### Entry into quarantine when the time of exposure is unknown

**Fig. 1:**
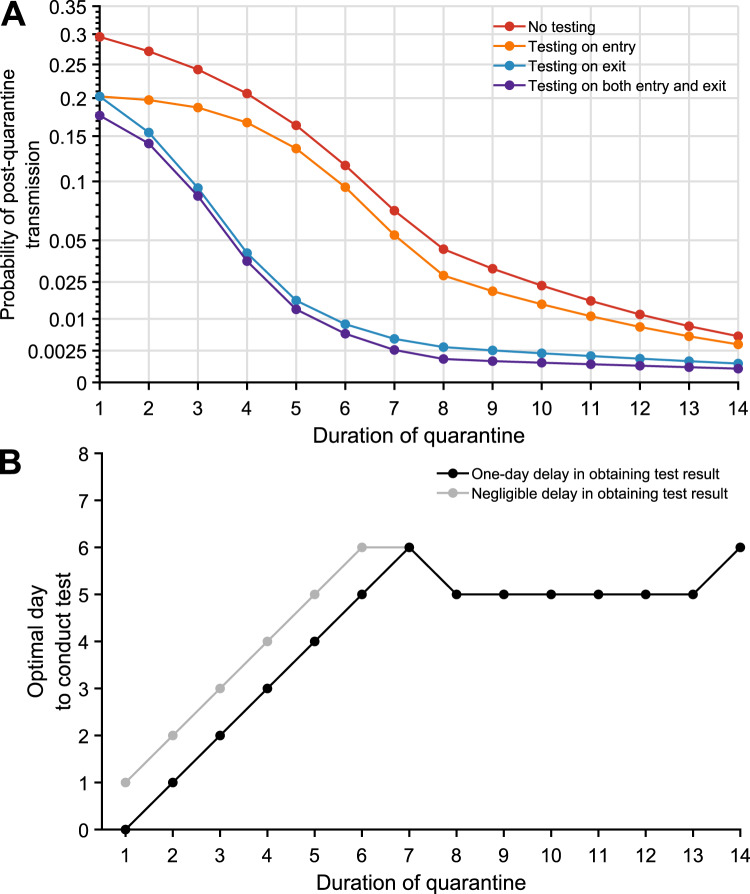
The impact of testing on the post-quarantine transmission for travel quarantine. The probability of post-quarantine transmission and optimal day to conduct the test when an infected individual enters quarantine uniformly within the incubation or asymptomatic period, for no testing and three testing strategies, and durations of quarantine from 1 to 14 days, with an incubation period of 8.29 days, 30.8% asymptomatic infections and perfect self-isolation of symptomatic infections. **A** Curves for the probability of post-quarantine transmission (one or more post-quarantine infections) without testing (red), with testing upon entry to quarantine (orange), on exit from quarantine (blue), and on both entry to and exit from quarantine (purple), incorporating with all testing strategies a one-day delay in sample collection to results, such that testing on exit occurred the day before the end of quarantine. **B** The optimal day to test during quarantine with a 1-day delay (black) and a negligible delay (gray) in obtaining test results.

For settings where there is no administrative knowledge of the time of exposure such as travel quarantine, we computed the expected PQT (Supplementary Fig. 2) and the probability of PQT after a range of quarantine durations without testing (Fig. 1A, Supplementary Fig. 3A). Assuming individuals self-isolate immediately upon symptom onset, the probability of PQT declines as the duration of quarantine increases (Fig. 1A). This probability is less than 0.25 with a quarantine duration of at least 3 days and falls below 0.05 for quarantines of eight days or longer.

The impact of quarantine can be augmented through testing. We assumed a 24-h delay between the sample collection and test results so that testing on exit occurred 1 day before the end of quarantine. Individuals who tested positive or developed symptoms were isolated until recovery. We found that any testing during quarantine contributed to a reduction in the probability of PQT across the full range of quarantine duration (Fig. 1A, Supplementary Fig. 3A). The magnitude of this reduction was dependent on both the duration of quarantine and the timing of the testing.

The largest reduction in the probability of PQT from conducting a single test occurred when it was performed on exit for quarantines of 7 days or less; on day five for quarantines lasting between eight and 13 days; and on day six for quarantines that are 14 days or longer (Fig. 1B, Supplementary Fig. 4A). As quarantined (asymptomatic) cases proceed through their quarantine, they simultaneously progress through their infectious course. Symptom onset will send a substantial fraction of infected individuals to isolation and diagnostic sensitivity decreases for the remainder^19^, leading to slightly diminishing benefits of “exit” tests performed later than day six.

Comparing the three testing strategies, we found that testing on both entry and exit from quarantine provides the greatest reduction in PQT, whereas the benefit of testing at entry is minimal (Fig. 1A, Supplementary Fig. 3A). Testing on exit consistently and substantially outperformed testing on entry across all quarantine durations considered (Fig. 1A).

We specifically compared strategies of quarantine and testing against the widely implemented WHO recommendation to quarantine for 14 days (without testing)^5^. In this comparison, a 13-day quarantine with testing on entry, a 7-day quarantine with testing on exit, and a 7-day quarantine with testing on both entry and exit each provide equivalent or lower probabilities of PQT (Fig. 1A, Supplementary Fig. 3).

### Assessment of quarantine and testing strategies implemented for offshore facilities

We applied our results in the context of employees of an off-shore oil company who were working a cycle of 26 days on, then 16 days off, a schedule that had been modified to make efficient use of a mandatory quarantine that was implemented during the pandemic. During the early stages of the epidemic, when prevalence was low, a 3-day quarantine had been implemented by the company in a secured facility, with testing on entry. Our risk-reduction models indicated substantial marginal benefit for increasing quarantine to 5–7 days with a test on exit. Testing on entry was retained for operational purposes, and testing 96 h later was initiated, accompanied by expansion to a 7-day quarantine for Region A and a 5-day quarantine for Region B.

**Fig. 2:**
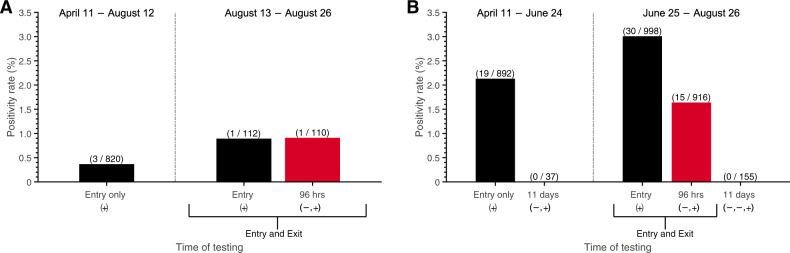
Positivity rates for testing on entry to and exit from quarantine. SARS-CoV-2 testing and positivity rate between April 11 to August 26, 2020, within two regions where crew members were quarantined: **A** region A, with a 7-day quarantine, where testing on entry (black) and exit (red) was started on August 13, and **B** region B, with a 5-day quarantine, where testing on entry (black) and exit (red) was started on June 25. Initially, a 3-day quarantine with testing only on entry was conducted in both regions. The vertical dashed line separates the early strategy of testing on only entry (left) and the later strategy of testing on both entry and exit (right), including follow-up post-quarantine tests conducted 11 days after the initial test (i.e., on day 12). Negative and positive sequential symbols − and + indicate the test histories. In these results, negative symbols are always conveying results to tests that were previous to the results quantified by the bar above. The number of positive tests (numerator) and the number of tests conducted (denominator) is denoted above the bar in parentheses.

To assess the practical implications of our recommendations, 4040 RT-PCR tests were conducted in region A and region B (serviced by different laboratories) prior to travel to offshore rigs. Among these, 69 results were positive (1.7%). Of the 1712 RT-PCR tests conducted on entry when the initial 3-day quarantine was in effect, there were 22 positive results (1.3%). After advisement, Region A deployed a 7-day home quarantine for all cycles starting August 13, where testing was performed on entry and exit (96 h after the first test); 50.0% (1/2) of the positive tests occurred on exit, following a negative test on entry (Fig. 2A). Starting June 25, Region B expanded to a 5-day hotel quarantine with testing on both entry and 96 h after the first test. For the period in which this strategy was implemented, 33.3% (15/45) of the positive tests were obtained upon the exit test, following a negative entry test (Fig. 2B). Further validation of the entry and exit testing protocol was provided through an additional 155 RT-PCR tests performed post-quarantine (11 days after the initial test) in Region B, all of which were negative.

No offshore worker registering negative tests on entry and on exit from quarantine was later diagnosed with COVID-19 during their offshore work. To quantify the added benefit of the test at 96 h, we calculated the probability of PQT for the cases detected by this second test. Compared with a 3-day quarantine and testing only on entry, extending the quarantine duration and adding testing on exit (96 h after the first test) reduced the probability of PQT by 98% for the 7-day quarantine and 93% for a 5-day quarantine. If the single case identified on the exit test from region A had remained undetected within the 7-day quarantine, we estimate an off-shore probability of PQT of 0.13. If the 15 cases that had been ascertained on exit from region B had remained undetected after the 5-day quarantine without testing on exit, we estimate that the probability of at least one event of PQT would have been 0.99, and would have resulted in an expected 9 offshore transmission events—each one serious concern for initiating further rapid spread and a disabling outbreak in the close quarters of an offshore rig.

### Accounting for the prevalence of the disease in the community

We evaluated the impact of disease prevalence in the community on the probability of PQT (Supplementary Fig. 6). For a cohort of 40 individuals undergoing a 5-day quarantine with a prevalence of 1%, we estimated the probability of PQT to be 0.06 for testing only on entry, and 0.005 for testing on both entry and exit (Supplementary Fig. 6B). For a 7-day quarantine and the same prevalence, the probability of PQT drops from 0.02 for testing only on entry to 0.001 when augmented with testing on exit (Supplementary Fig. 6C).

### Contrasting contact tracing and uniform entry into quarantine

**Fig. 3:**
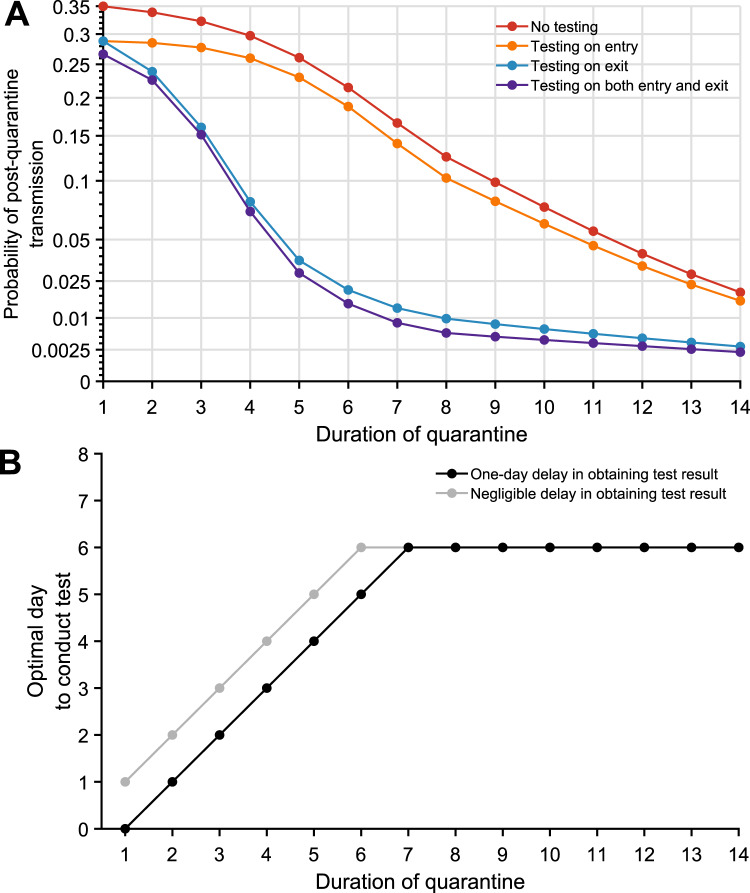
The impact of testing on the post-quarantine transmission for traced contacts. The probability of post-quarantine transmission for no testing and three testing strategies applied to 1–14-day durations of quarantine, when an individual enters quarantine through contact tracing, specifying an incubation period of 8.29 days, 30.8% asymptomatic infections, and perfect self-isolation of symptomatic infections. **A** Curves for the probability of post-quarantine transmission (one or more post-quarantine infections) without testing (red), with testing upon entry to quarantine (orange), on exit from quarantine (blue), and on both entry to and exit from quarantine (purple), incorporating with all testing strategies a 1-day delay in sample collection to results, such that testing on exit occurred the day before the end of quarantine. **B** The optimal day to test during quarantine for a specified quarantine duration, with a 1-day delay (black) and with a negligible delay (gray) in obtaining test results.

Contact tracing is ideally initiated following the identification of a positive case either by symptom presentation or by surveillance screening through testing. We evaluated the impact of quarantine initiated through contact tracing on reducing PQT under scenarios of no delay (Fig. 3A, Supplementary Fig. 7, Supplementary Fig. 8) or 1-day delay in outreach to exposed contacts (Supplementary Fig. 9, Supplementary Fig. 10). Tracing of contacts was assumed to be initiated by the onset of relevant COVID-19 symptoms in the index case. Rapid contact tracing results in the quarantine of infected contacts early in their infection course, thereby increasing the recommended duration of quarantine and changing the relationship between test timing and the probability of PQT, compared to uniform entry into quarantine (Fig. 3A vs. Fig. 1A).

However, the combination of shorter quarantines with exit testing maintains high effectiveness compared with 14-day quarantines without testing. When cases are identified through contact tracing, we found that a 7-day quarantine with testing on exit and a 6-day quarantine with testing on entry and exit each result in a probability of PQT equivalent or lower than a 14-day quarantine with no testing; testing on entry bestowed only trivial benefit (Fig. 3A, Supplementary Fig. 8). For quarantines of 7 days or less, the optimal test timing was upon exit. For quarantines beyond 7 days, the optimal timing was day six (Fig. 3B, Supplementary Fig. 4B).

### Optimal day of testing for a known time of exposure

**Fig. 4:**
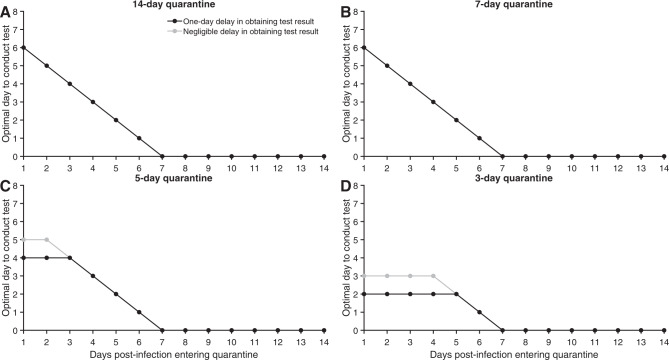
Optimal testing day for a known time of exposure. For a case whose date of exposure has been identified as occurring 1–14 days prior to quarantine, the optimal day to conduct the RT-PCR test with a 1-day delay (black) and with a negligible delay (gray) in obtaining test results, assuming perfect self-isolation of symptomatic infections, 30.8% asymptomatic infections, an incubation period of 8.29 days, and a quarantine lasting **A** 14 days, **B** 7 days, **C** 5 days, and **D** 3 days.

When a specific date of exposure can be identified for a traced contact, the optimal test timing differs from that calculated by integrating over all possible exposure times. With a 14-day quarantine starting 1 day post-infection, we estimated that testing on day six of quarantine is optimal; with quarantine starting later, the optimal day of testing then decreased linearly. For shorter quarantines, we found testing on exit to be optimal for individuals entering early in the disease time course. For an individual entering quarantine seven or more days post-infection, the optimal test date is the test on entry (Fig. 4).

### Sensitivity analyses

We performed a comparative analysis specifying a latent period that is 1 day greater or lesser than the reported 2.9 days^22^. The expected number of secondary cases occurring before symptom onset was similar among the different latent periods (1.21 infection for a latent period of 2.9 days; 1.24 infections for a latent period of 1.9 days; and 1.27 infections for a latent period of 3.9 days). The infectivity profiles differed among the three latent periods, with peak infectivity that is higher for both the 1.9-day and 3.9-day latent periods when compared to our baseline (Supplementary Fig. 11).

For quarantine periods of at least seven days and individuals entering quarantine uniformly across the time course of infection (Supplementary Figs. 12–15), the probability of PQT was lower for shorter latent periods. For shorter quarantines, the relationship between the probability of PQT and latent period is more intricate. For traced contacts entering quarantines of 8 days or longer (Supplementary Figs. 16–19), shorter latent periods entailed a lower probability of PQT. For traced contacts entering quarantines of fewer than 8 days, the relationship of the latent period to the probability of PQT is more complex. However, 1-day changes in the latent period affect the optimal day to conduct a single test by at most 1 day (Supplementary Fig. 4). Specifically, we found that a 3.9-day latent period decreased the optimal day of testing estimated for a 2.9-day latent period, whereas a 1.9-day latent period increased the best day to conduct a single test.

For our analysis of potential outbreaks consequent to offshore-rig quarantine and testing, we analyzed the sensitivity of our result to the proportion of asymptomatic individuals on the probability of PQT (Supplementary Fig. 5). We found that the estimated probability of PQT using the strategy of testing upon entry and at 96 h moderately increased if a higher proportion of infections were expected to be asymptomatic (Supplementary Fig. 5).

## Discussion

Here, we derived theory to calculate the probability of PQT of COVID-19 for a wide range of durations of quarantine, supplemented by testing on entry to quarantine, on exit from quarantine, or both. For quarantines with durations of up to 7 days, we found that testing on exit provided the greatest marginal benefit in terms of reducing the probability of PQT. Testing on entry provided modest benefits in combination with quarantine or with testing on exit. For a quarantine with a duration longer than 7 days, the optimal testing time is on day five or six. Optimal testing times were fairly consistent between travel quarantines and quarantines of traced contacts, differing at most by a day. The benefits of testing later in quarantine were demonstrated by test results of oil crewmembers heading offshore that identified 16 cases testing negative on entry and positive on exit that could easily have resulted in costly and logistically difficult-to-handle offshore outbreaks. When the time of exposure is known, the optimal day for a test for quarantines of a week or more starts at day six of the quarantine, decreasing linearly to day-of-entry for individuals who have been infected for seven or more days. It may seem counter-intuitive that the optimal test for so many identified timings of exposure is on entry, yet testing on entry has so much less impact than testing on exit when the date of exposure is unknown. Indeed, for individuals that are tested after the incubation period (e.g., later than symptom onset), the diagnostic sensitivity of the RT-PCR test has started to decline. However, for individuals late in the disease, there is also far less infectivity left in their disease course. The high remaining infectivity of individuals early in the disease course markedly outweighs the low infectivity of individuals late in the disease course in influencing the optimal day of testing to prevent PQT.

An outbreak can be triggered or sustained within an environment that is monitored only for symptoms of COVID-19. Quarantining individuals before returning to work or school has been a common strategy among many businesses, the military, and universities to prevent potential outbreaks^23,24^. An offshore or military setting is one of the numerous close-quarters environments in modern society where an outbreak can seriously impact operational integrity, leading to compromised safety and adverse economic consequences. Hence, minimizing outbreak risk while maintaining staffing is critical. Testing may allow for the quarantine duration to be reduced without increasing the risk of PQT. For example, many universities have implemented plans for quarantining and frequent testing of students and employees, where resources allow^25,26^. For businesses and close-quarters environments, the impact of false negatives is a substantially greater issue for operational integrity than false positives. Consistent with the results from our analytic model (Fig. 1A and Fig. 3A), simulations from a recent agent-based model suggest that testing on exit—or entry and exit—of a 7-day quarantine can avert similar transmission as a 14-day quarantine with no transmission^12^. Our results show that testing upon entry to quarantine carries such a risk of false negatives, as infected individuals who enter quarantine very early in the incubation period of the disease may not be detected due to low viral loads.

Our estimates for the probability of PQT for the various strategies were estimated assuming a basic reproductive number of 2.5 throughout the disease course, and unchanged post-quarantine. In the offshore environment, individuals are living in very confined quarters which could lead to higher PQT and a larger number of secondary infections. In some community settings, the number of secondary infections can be reduced through mask-wearing, social distancing, and other non-pharmaceutical interventions. These changes in the number of secondary infections post-quarantine can markedly influence the probability of PQT. However, they would not affect the relative benefit of testing on exit compared to entry. Therefore, our qualitative finding of the optimality of testing later in quarantine than on entry are robust to settings with extensive PQT.

As prevalence in the general community increases (Supplementary Fig. 6, blue and purple), there are benefits to conducting additional tests during quarantine: as substantial numbers of infected individuals enter quarantine, larger numbers of individuals may proceed through testing with rare false-negative test results, increasing PQT. Addressing false negatives that inevitably occur at high prevalence can be aided by performing additional tests during quarantine; the impact of any specific set of tests can be quantified within our model framework. In future research, the theory can be applied to evaluate the impact of incorporating recent innovations such as saliva RT-PCR tests and rapid antigen tests. These alternate approaches could exhibit altered optima. We have not quantified more extensive testing strategies here due to the limited availability of testing, potentially high and largely unknown correlations among false-negative test results for individual cases, and the observed moderate marginal benefit of additional testing performed in the early stages of the disease with lower detection rates (Supplementary Fig. 28).

Optimal timing of limited testing during quarantine improves the ability to control PQT. Testing several days into quarantine increases the probability of an infected case testing positive (Supplementary Fig. 4). The increasing diagnostic sensitivity of the RT-PCR test is attributable to the rapidly increasing viral load following the less detectable latent stage of infection. If the infected individual remains asymptomatic, testing near the end of a standard 14-day quarantine can also lead to low diagnostic sensitivity due to a declining viral load as they overcome the infection^27^. Australia has implemented a mandatory 14-day quarantine for individuals arriving into the country, with testing during the first 2 days of arrival and between days 10 and 12 of quarantine^16^. Though the differences are moderate, our analysis indicates that the lowest probability of PQT is achieved by testing on day six of the standard 14-day quarantine (Fig. 1B, Fig. 3B).

Testing was found to result in a smaller reduction of the expected PQT when cases enter quarantine through contact tracing compared to when they enter as a consequence of travel regulation. Contact tracing will usually identify more infected cases per quarantined individual than will travel quarantine, due to the specific exposure risk. For example, if prevalence is 1% and 10 individuals are selected at random for quarantine, then on average 0.1 people would be infected. Alternatively, if an index case is isolated upon symptom onset, there would be on average 1.21 individuals infected (for an *R*_0_ = 2.5) prior to symptom onset and potentially identified through contact tracing. With a significant chance of traced contacts being infected, reducing PQT becomes increasingly important. However, traced contacts are likely to enter quarantine earlier in disease (Supplementary Fig. 31). Such an earlier entry necessitates a consequently longer quarantine (generally). The earlier entry makes it more likely that testing early in quarantine will occur during the latent period when the diagnostic sensitivity of the RT-PCR test is highly limited.

Our study is informative for businesses, military operations, and universities, providing a quantitative estimation of the residual risk of PQT. The calculated infection risks were used to inform the quarantine and RT-PCR testing strategy deployed by an oil and gas company prior to workers traveling offshore. Of the positive tests obtained under this strategy, 34% were obtained on an exit test following a negative entry test. The exit test prevented 16 infected crew members from exiting quarantine and entering confined quarters offshore while potentially infectious. The results of the time of testing for a given quarantine duration are also useful for public-health decision making when quarantine is required for international, interstate, and social travel.

Our examination of the effects of duration of quarantine and timings of testing is critical to future efforts to balance the risk of PQT with the economic costs, negative impact on mental health, and restrictions on social liberty associated with prolonged quarantines. Timely testing enables a shorter quarantine with equivalent benefits to the much longer 14-day quarantine in the prevention of PQT. Our study indicates that the strategy of testing upon entry into quarantine—currently implemented by many institutions and administrative bodies—conveys the least benefit if infection time is unknown. Testing at the exit can provide substantially higher dividends in reducing PQT; or at an optimal timing near one week for quarantines of a week or longer. Our result was substantiated both by our integrative analysis of infectivity and diagnostic sensitivity and by test results demonstrating the utility of tests 96 h into the quarantine of crew members of an offshore oil facility. In determining policies for the duration of travel quarantine and quarantine of traced contacts, full consideration of how timely diagnostic testing aids prevention of PQT is essential to the effective and transparent balancing of lives and livelihoods in times of a global pandemic.

## Methods

### Epidemiological parameters

The average incubation period is 8.29 days^18^. The latent period (i.e., infected but low probability of infecting contacts) is 2.9 days^22^. We consider latent periods of 1.9 days and 3.9 days in a scenario analysis^22^ (Supplementary Figs. 11–19).

For our baseline analysis, we considered a delay of one day between sample collection and the result of the RT-PCT test. Thus, the sample is taken 1 day before the end of quarantine when testing on exit. We also conducted the analysis when there was no delay in testing results to examine the impact on the probability of PQT (Supplementary Figs. 20–23).

In the baseline analysis, we assumed *R*_0_ = 2.5 and 30.8% of infections are asymptomatic^8,20^. We further analyzed the scenario in which 22.6% of infections are asymptomatic (Supplementary Figs. 24–27)^28^. Both of these proportions are consistent with estimates from a systematic meta-analysis^21^. Asymptomatic infections were assumed to be equally as infectious as symptomatic infections. This assumption is based on measurements of viral loads in asymptomatic infections being comparable to those observed in symptomatic cases^29,30^.

### Infectivity profile

The infectivity profile has been determined to increase rapidly prior to symptom onset, a peak near the onset of symptoms, and decrease subsequently^31^. We specified our infectivity profiles based on the full dataset and R code provided by He et al.^17^, specifying the latent period. The infectivity during the latent period was expressed as exponentially lower (Supplementary Methods: Infectivity function). Imposing the strict threshold where 20 days after symptom onset infectivity is zero^32–34^ made no significant difference to our estimate of PQT for quarantines of up to 14 days.

### Temporal diagnostic sensitivity of a SARS CoV-2 RT-PCR assay

We utilized the post-symptom onset temporal diagnostic sensitivity for RT-PCR tests of infected individuals^19^, fitting a logistic regression function to the diagnostic sensitivity data (obtained through digitization with WebPlotDigitizer^35^) from zero to 25 days post-symptom onset through minimization of least squares. To infer the diagnostic sensitivity prior to symptom onset, we first used this function to perform a slight extrapolation of the diagnostic sensitivity back to the peak, which occurred slightly prior to symptom onset. Second, to determine the diagnostic sensitivity for the remaining portion of the incubation period, we specified the interpolation function determined by the infectivity and the diagnostic sensitivity from the post-symptom onset and used that interpolation function on the pre-symptom onset infectivity to determine pre-symptom onset diagnostic sensitivity (Supplementary Methods: Diagnostic sensitivity function). This process provides diagnostic sensitivity over the entire course of infection (Supplementary Fig. 28). We assumed that the specificity of the RT-PCR assay was 100%^36^.

### Probability of PQT

To calculate the probability of PQT—defined to be the probability of at least one post-quarantine infection—we assumed that the expected PQT is described by a negative binomial distribution with a dispersion parameter of 0.25^37^. This value for the dispersion parameter is consistent with numerous published estimates^38–40^. For sensitivity analyses, we also computed the probability of PQT given Poisson-distributed PQT (Supplementary Figs. 29 and 30). In our additional analysis accounting for the underlying prevalence within the community, the probability of PQT was defined as the likelihood that at least one infected individual in a cohort became a source of PQT. Similarly, to calculate the probability of PQT given a negative test on the entry for *N* infected individuals, we estimated the probability that at least one of the cases contributed to PQT.

### Data of SARS CoV-2 tests during the quarantine

Between April 11, 2020 and August 26, 2020, there were 4040 SARS CoV-2 RT-PCR tests conducted among employees of an oil and gas company coming from two regions (stratified by lab location). A third region that was monitored is not included in our data set, as there was low population prevalence entering quarantine and there were no positive tests. During the early stages of the epidemic, both regions used a 3-day quarantine with testing on entry. On August 13, employees from region A quarantined at home for 7 days, with testing occurring on both entry and exit. While employees were at home, they were asked to practice social distancing in public. Starting on June 25, employees from region B were quarantined in a hotel for 5 days prior to their departure offshore and tested on both entry and exit. The requirements of an employee to go off-shore were (1) passing the components of a screening form used to filter out symptomatic cases and those potentially exposed, (2) temperature screenings, and (3) completion of the quarantine with no positive RT-PCR test. Upon a positive test, the employee initiated a 14-day isolation period and followed through with the company’s case management process. After the isolation period, individuals were able to return back to work contingent upon two negative RT-PCR tests. The crew members were 97% male, ranging from 24 to 64 years of age (average age 34 years). Each individual provided informed consent and signed a HIPPA release for the test ordered by XstremeMD, the medical group that collected the tests. The use of this data was approved by the Human Participants Review Sub-Committee, York University’s Ethics Review Board (Certificate Number: 2020-323).

### Reporting summary

Further information on research design is available in the Nature Research Reporting Summary linked to this article.

## Supplementary information

Supplementary Information

Reporting Summary

## Data Availability

The number of positive tests and tests conducted at the two regions quarantining the crew members heading offshore is presented in Fig. 2, with other data used in the analysis referenced in Supplementary Table 1 and in the Methods.

## Code availability

The computational code for the analysis was implemented in MATLAB, and it is available online^41^.
