# Supplementary material for: Optimal COVID-19 quarantine and testing strategies
Source: Nat Commun. 2021 Jan 7;12:356. doi: 10.1038/s41467-020-20742-8 (PMC7788536; doi:10.1038/s41467-020-20742-8)
Supplement: Supplementary file 1 — Supplementary Information [file 41467_2020_20742_MOESM1_ESM.pdf]

# Supplementary Information:
# Optimal COVID-19 quarantine and testing strategies

Chad R. Wells[1], Jeffrey P. Townsend[2,3,4,5]*, Abhishek Pandey[1], Seyed M. Moghadas[6], Gary Krieger[7,8], Burton Singer[9], Robert H. McDonald[10], Meagan C. Fitzpatrick [1,11], Alison P. Galvani[1,3]

[1] Center for Infectious Disease Modeling and Analysis (CIDMA), Yale School of Public Health, New Haven, Connecticut 06520, USA
[2]Department of Biostatistics, Yale School of Public Health, New Haven, Connecticut 06510, USA
[3]Department of Ecology and Evolutionary Biology, Yale University, New Haven, Connecticut 06525, USA
[4]Program in Computational Biology and Bioinformatics, Yale University, New Haven, Connecticut 06511, USA
[5]Program in Microbiology, Yale University, New Haven, Connecticut 06511, USA
[6] Agent-Based Modelling Laboratory, York University, Toronto, Ontario, M3J 1P3, Canada
[7] NewFields E&E Boulder, Colorado 80301, USA
[8] Skaggs School of Pharmacy and Pharmaceutical Science, University of Colorado Anschutz Medical Campus, Aurora, Colorado 80045, USA
[9] Emerging Pathogens Institute, University of Florida, P.O. Box 100009, Gainesville, Florida 32610, USA
[10] Group Health, Safety and Environment; BHP; Melbourne, Victoria, Australia, 3000
[11] Center for Vaccine Development and Global Health, University of Maryland School of Medicine, Baltimore, Maryland, 21201, USA

These authors contributed equally: Chad R. Wells and Jeffrey P. Townsend

* Corresponding author: Jeffrey.Townsend@Yale.edu

# Supplementary Methods

## *Transmission over Time*

Transmission of a pathogen from an infected individual is typically time-dependent, based on pathogen shedding and behavioral changes, and can be represented over time by a function $r(t)$, for which time $t$ = 0 represents initial infection. To represent infectiousness, a function $r(t)$ can be scaled such that

$$\int_{t=0}^{\infty} r(t)\mathrm{d}t = R_0, \qquad (1)$$

where $R_0$ is the basic reproduction number: the expected number of infections consequent to a single infected individual under a scenario of no intervention. Specifying a discrete end to the infection at time $t_e$ such that $r(t) = 0$ for $t > t_e$,

$$\int_{t=0}^{t_e} r(t)\mathrm{d}t = R_0.$$

Infectiousness during discrete timespans $t_2 - t_1$ (e.g. days) can be calculated as

$$R_{t_2-t_1} = \int_{t=t_1}^{t_2} r(t)\mathrm{d}t.$$

## *Self-isolation at Symptom Onset*

A significant means of intervention to prevent infection is self-isolation of infected individuals upon symptom onset. The expected effect on onward transmission of an intervention such as self-isolation of a case that becomes symptomatic at time $t_s$ can be calculated as

$$R_i = \int_{t=0}^{t_s} r(t)\mathrm{d}t, \qquad (2)$$

provided that all individuals self-isolate upon presentation with symptoms. If $\int_{t=0}^{t_s} r(t)\,\mathrm{d}t > 1$, then even perfect self-isolation upon symptom onset will be insufficient to extinguish disease transmission. We express the transmission over time for a symptomatic individual who isolates upon symptom onset as

$$r_S(t) = \begin{cases} r(t) & 0 \leq t \leq t_s, \\ 0 & t > t_s \end{cases} \quad .$$

If the outcome of infections leads to a proportion of infected individuals $p_a$that can infect others but that never manifest symptoms (i.e. that are asymptomatic carriers), then transmission may be partitioned into the contributions of symptomatic and asymptomatic cases as $R_0 = R_{0,s}p_s + R_{0,a}p_a$, in which the probability of a symptomatic case $p_s = (1 - p_a)$. $R_{0,s}$ and $R_{0,a}$ can be equated to distinct infectiousness functions $r_s(t)$ and $r_a(t)$, in the absence of self-isolation. For simplicity of presentation in ensuing theory, it will be assumed that $R_{0,s} = R_{0,a}$ and the same infectivity profile in the absence of self-isolation (i.e. $r_s(t) = r_s(t) = r(t)$) [1,2]. Alternate overall transmission and alternate forms of infectivity over time for asymptomatic cases may easily be partitioned and tracked in the theory that follows should there be evidence to substantiate their difference.

The presence of asymptomatic carriers increases the degree of transmission consequent to a self-isolation intervention from that shown by Eq. 2 to

$$R = p_s \int_{t=0}^{t_s} r_S(t)\,\mathrm{d}t + p_a R_0 .$$

***Quarantine***

**Quarantine with a Known Time of Infection.** A longstanding approach to limit disease spread is the quarantine of individuals who have no prior indication of potential for disease but intend to migrate from a population in which there is current transmission to a population with lower or zero disease prevalence. Because quarantined individuals experience a significant restriction of personal freedom, it is important to minimize the duration of quarantine $q$, but also maximize its effectiveness in limiting post-quarantine transmission. Quarantine of $q$ days from time $t_q$to time $t_q + q$ limits total expected post-quarantine transmission to

$$R_q = R_0 - \int_{t_q}^{t_q + q} r(t)\,\mathrm{d}t.$$

For policy decision-making regarding quarantine duration, the expected post-quarantine transmission is typically most important, and can be calculated as

$$R_{q\mapsto} = \int_{t\,=\,t_q+q}^{\infty} r(t)\,\mathrm{d}t.$$

If individuals self-isolate, there is a trivial case in which $t_s \leq t_q + q$ and $R_{q\mapsto} = 0$; otherwise, $t_s > t_q + q$ and

$$R_{q\mapsto} = \int_{t\,=\,t_q+q}^{\infty} r_S(t)\,\mathrm{d}t.$$

Including asymptomatic carriers,

$$R_{q\mapsto} = p_s \int_{t\,=\,t_q+q}^{\infty} r_S(t)\,\mathrm{d}t + p_a \int_{t\,=\,t_q+q}^{\infty} r(t)\,\mathrm{d}t.$$

Unfortunately, these expressions are unlikely to be useful in this form for quantifying the benefits of quarantine in reducing transmission. In the case of quarantine of migrants from one population to another, the time of infection—and correspondingly the time of quarantine $t_q$—are rarely known.

**Quarantine with an Unknown Time of Infection.** In a rapidly spreading epidemic, individuals who might be entering quarantine will tend to be early in disease time course. In a rapidly declining epidemic, individuals who might be entering quarantine will tend to be later in disease time course. In a steady-state epidemic with case counts $c(t)$, $\frac{dc}{dt} \approx 0$ over the period from $t_0$ to $t_s$ such that individuals entering quarantine are evenly distributed across the disease time course. Provided all individuals experience symptoms at time $t_s$ that qualify them for isolation instead of quarantine, then the expected post-quarantine infectivity is

$$r_q(t) = \frac{1}{t_s} \int_{u=\,0}^{t_s} r_S(t+u)\,\mathrm{d}u,$$

and expected post-quarantine transmission from an infected individual is

$$R_{q\mapsto}(q) = \frac{1}{t_s} \int_{u=0}^{t_s} \int_{t=u+q}^{\infty} r_S(t)\,\mathrm{d}t\,\mathrm{d}u\ ,$$

a function of days of quarantine $q$. For asymptomatic carriers entering within disease time course $t_e$,

$$R_{q\mapsto}(q) = \frac{1}{t_e} \int_{u=0}^{t_e} \int_{t=u+q}^{\infty} r(t)\,\mathrm{d}t\,\mathrm{d}u.$$

Incorporating both symptomatic and asymptomatic infections,

$$R_{q\mapsto}(q) = \frac{p_s}{t_s} \int_{u=0}^{t_s} \int_{t=u+q}^{\infty} r_S(t)\,\mathrm{d}t\ \mathrm{d}u \ + \frac{p_a}{t_e} \int_{u=0}^{t_e} \int_{t=u+q}^{\infty} r(t)\,\mathrm{d}t\,\mathrm{d}u.$$

A similar approach that incorporates symptomatic and asymptomatic cases by their proportions within the population may be performed throughout the rest of the scenarios below, and will not be specifically pointed out for each scenario.

***Testing***

**Testing with a Known Time of Infection.** Diagnostic test sensitivity $s(t)$ is also time-dependent. Assaying for components of the pathogen (e.g. DNA, RNA, or protein), diagnostic sensitivity typically is zero to low very early in disease before the pathogen load burgeons, then declines in the later stages of disease when immune responses develop and infection is suppressed (Supplementary Fig. 28). In a disease for which tests can diagnose infections during the incubation phase, testing can enhance the efficacy of quarantine by identifying individuals to be isolated instead of quarantined, thereby preventing future transmission from cases that persist as infectious through an earlier exit from quarantine than would be called for in case isolation.

**Testing with an Unknown Time of Infection.** The temporal diagnostic sensitivity of a test for infected cases with an unknown time of infection can be calculated by integrating over the

unknown time of infection, such that

$$s_u(t) = \frac{1}{t_e}\int_{u=0}^{t_e} s(t+u)\ \mathrm{d}u.$$

***Quarantine and Testing***

**Quarantine with an Unknown Time of Infection with Testing on Entry**. Assuming the duration of the quarantine, $q$, is longer than the delay between administering the test and acting to isolate upon a positive result, the expected post-quarantine infectivity over time of a symptomatic individual whose time of infection is unknown and who is tested for disease on entry to quarantine is

$$r_{q^{\mapsto}}(t) = \frac{1}{t_s}\int_{u=0}^{t_s}\big(1 - s(u)\big)\cdot\ r_S(t+u)\ \mathrm{d}u,$$

in terms of time from infection. In terms of $q$ days of quarantine, the expected post-quarantine transmission is

$$R_{q^{\mapsto}}(q) = \frac{1}{t_s}\int_{u=0}^{t_s}\int_{t=q}^{\infty}\big(1 - s(u)\big)\cdot\ r_S(t+u)\ \mathrm{d}t\ \mathrm{d}u.$$

For asymptomatic carriers,

$$R_{q^{\mapsto}}(q) = \frac{1}{t_e}\int_{u=0}^{t_e}\int_{t=q}^{\infty}\big(1 - s(u)\big)\cdot\ r(t+u)\ \mathrm{d}t\ \mathrm{d}u\ .$$

**Quarantine with an Unknown Time of Infection with Testing on Entry and Exit.** Expected post-quarantine transmission from an individual whose time of infection is unknown and who is tested for disease upon entry and at the last opportunity prior to the end of quarantine is

$$R_{q^{\mapsto}}(q) = \frac{1}{t_s}\int_{u=0}^{t_s}\int_{t=q}^{\infty}\big(1 - s(u)\big)\cdot\big(1 - s(u+q-d_t)\big)\cdot\ r_S(t+u)\ \mathrm{d}t\ \mathrm{d}u\ ,$$

where $d_t$ is the delay between administering the test and isolation if positive. For asymptomatic carriers,

$$R_{q^{\mapsto}}(q) = \frac{1}{t_e}\int_{u=0}^{t_e}\int_{t=q}^{\infty}\big(1 - s(u)\big)\cdot\big(1 - s(u+q-d_t)\big)\cdot\ r(t+u)\ \mathrm{d}t\ \mathrm{d}u.$$

**Quarantine with Testing at Any Time(s).** Expected post-quarantine transmission of an infected individual whose time of infection is unknown and who is tested for disease at any time $0 \le t_t \le q - d_t$ is

$$R_{q\mapsto}(q) = \frac{1}{t_s}\int_{u=0}^{t_s}\int_{t=q}^{\infty}\left(1 - s(t_t + u)\right) \cdot r_S(t+u)\,\mathrm{d}t\,\mathrm{d}u.$$

For asymptomatic carriers,

$$R_{q\mapsto}(q) = \frac{1}{t_e}\int_{u=0}^{t_e}\int_{t=q}^{\infty}\left(1 - s(t_t + u)\right) \cdot r(t+u)\,\mathrm{d}t\,\mathrm{d}u.$$

Additional terms $\left(1 - s(u + t_k)\right)$, where $k$ indexes testing times, may be included as terms within the product inside the double integral to quantify the expected post-quarantine transmission of any schedule of testing to be applied during quarantine.

**Quarantine with a Negative Test on Entry**. The probability density for obtaining a false negative upon entry for a symptomatic individual is

$$f_S(t) = \begin{cases} \frac{1-s(t)}{\int_{u=0}^{t_s} 1-s(u)\mathrm{d}u}, & \text{if } 0 \le t \le t_S, \text{ and} \\ 0, & \text{otherwise} \end{cases},$$

and the probability density for an asymptomatic individual is

$$f_A(t) = \begin{cases} \frac{1-s(t)}{\int_{u=0}^{t_e} 1-s(u)\mathrm{d}u}, & \text{if } 0 \le t \le t_e, \text{ and} \\ 0, & \text{otherwise.} \end{cases}$$

The expected post-quarantine infectivity over time of a symptomatic individual who tested negative for disease on entry to quarantine is

$$r_{q\mapsto}(t) = \int_{u=0}^{t_s} f_S(u) \cdot r_S(t+u)\,\mathrm{d}u,$$

in terms of time from infection. In terms of $q$ days of quarantine, the expected post-quarantine transmission is

$$R_{q\mapsto}(q) = \int_{u=0}^{t_s}\int_{t=q}^{\infty} f_S(u) \cdot r_S(t+u)\,\mathrm{d}t\,\mathrm{d}u.$$

For asymptomatic carriers, the expected post-quarantine infectivity is

$$r_{q\mapsto}(t) = \int_{u=0}^{t_e} f_A(u) \cdot\ r(t+u)\ \mathrm{d}u,$$

and the expected post-quarantine transmission is

$$R_{q\mapsto}(q) = \int_{u=\ 0}^{t_e} \int_{t=q}^{\infty} f_A(u) \cdot\ r(t+u)\ \mathrm{d}t\ \mathrm{d}u.$$

***Contact Tracing***

Tracing of individuals who have had contact with an index case identifies persons whose quarantine would reduce the risk of disease transmission from recently exposed individuals. When an individual is identified as a contact of an index case, the expected time of infection is not the same as that of an individual selected at random from an infected population. Restricting our attention to transmissions occurring between an index case and their contacts, there are four nominal transmission relationships to be considered, of which three are considered relevant to an attentive program of contact tracing and quarantine (Supplementary Table 2): the asymptomatic or pre-symptomatic contact may have infected the index case, or may have been infected by the index case. Here we excluded from calculation the case in which a pre-symptomatic individual infects the index case, because that scenario is formally impossible with a fixed $t_s$ and rigorous self-isolation and self-identification upon symptoms, and unlikely even with variable $t_s$ and imperfect adherence to self-isolation and self-identification.

**Supplementary Table 2.** Modeled infectivity functions for the contact identified during tracing

| Contact | Infected by the index case | Infected the index case |
|---|---|---|
| Symptomatic case | $r_{I\to S}(t)$ | — |
| Asymptomatic carrier | $r_{I\to A}(t)$ | $r_{A\to I}(t)$ |

**A To-be-Symptomatic Contact Infected by the Index Case but not yet Symptomatic.** By assumption, infection of the contact must have occurred prior to the onset of symptoms in the index case. The likelihood that an infection from the index case occurred at a time during the disease time course of the index case should proportionally follow $r(t)$ (Eq. 1). Thus, the probability density for infection—on the timescale *t* of the infection of the index case that was identified at symptom onset—is

$$\iota(t) = \begin{cases} \frac{r_S(t)}{\int_{x=0}^{t_s} r_S(x)\mathrm{d}x}, & \text{if } 0 \leq t \leq t_S, \text{ and} \\ 0, & \text{otherwise.} \end{cases}$$

The probability density for the time since infection of the to-be-symptomatic contact—on the timescale *t* of the contact—is

$$\eta(t) = \iota(t_s - t).$$

Thus, the erstwhile expected infectivity from the contact that was infected by the index case from the time of intervention by a quarantine is

$$r_{I\to S}(t)=\int_{v=0}^{t_s} \eta(v) \cdot r_S\left(v + t + d_q\right) \mathrm{d}v,$$

where $d_q$ is the delay from identifying the index case to quarantine of the contact. The expected post-quarantine transmission by the contact after a quarantine of duration $q$ is

$$R_{I\to S_q\mapsto}(q) = \int_{v=0}^{t_s} \int_{w=q+d_q}^{\infty} \eta(v) \cdot r_S(v + w) \,\mathrm{d}v \,\mathrm{d}w.$$

**An Asymptomatic Carrier Contact Infected by the Index Case.** The expected infectivity of an asymptomatic contact infected by the index case—from time $t = 0$ at intervention by quarantine—is

$$r_{I\to A}(t)=\int_{v=0}^{t_s} \eta(v) \cdot r\left(v + t + d_q\right) \mathrm{d}v,$$

where $d_q$ is the delay from identifying the index case to quarantine of the contact. The expected post-quarantine transmission from the asymptomatic contact infected by the index case starting from the time of intervention by a quarantine of duration $q$ is

$$R_{I\to A_q\mapsto}(q) = \int_{v=0}^{t_s}\int_{w=q+d_q}^{\infty}\eta(v)\ \cdot r(v+w)\ \mathrm{d}v\ \mathrm{d}w.$$

**An Asymptomatic Contact that Infected the Index Case.** Because the index case was assumed to be identified due to symptom onset, an asymptomatic contact that infected the index case must have already been infected for a duration of at least $t_s + d_q$. Consequently, the probability density of infection from that contact is

$$\kappa(t) = \begin{cases} \frac{r(t)}{\int_{s=t_s+d_q}^{\infty} r(s)\mathrm{d}s}, & \text{if } t \geq t_S + d_q, \text{ and} \\ 0, & \text{otherwise.} \end{cases}$$

Setting $K = \int_{v=t_s+d_q}^{\infty} r(v)\ dv$, the expected infectivity of the asymptomatic contact that infected the symptomatic index case—from time $t$ = 0 at intervention by quarantine—is

$$r_{A\to I}(t) = \frac{1}{K}\int_{v=t_s+d_q}^{\infty} r(v)\ \cdot r(t+v)\ \mathrm{d}v,$$

and the expected post-quarantine transmission is

$$R_{A\to I_q\mapsto}(q) = \frac{1}{K}\int_{w=q}^{\infty}\int_{v=t_s+d_q}^{\infty} r(v)\cdot r(w+v)\ \mathrm{d}v\ \mathrm{d}w.$$

Continuing our assumption that individuals are assiduously self-isolating upon symptom onset and recalling that $R_i = \int_{t\,=\,0}^{t_s} r_S(t)\mathrm{d}t$ (Eq. 2), we can tabulate the expected transmission by contacts of the index that are classified into three kinds (Supplementary Table 3). By assumption, a contact to become symptomatic could not have infected the index case, because otherwise in an assiduously self-isolating population, that contact would have been the index case.

**Supplementary Table 3.** Expected infections from contacts of each modeled transmission type.

| Contact | Infected by the index case | Infected the index case |
|---|---|---|
| Symptomatic case | $p_s R_i$ | — |
| Asymptomatic carrier | $p_a R_i$ | $p_a R_o$ |

Combining all three transmission functions of contacts of an index case discovered due to appearance of symptoms, the expected post-quarantine infectivity

$$r_c(t) = \frac{p_s R_i}{R_i + p_a R_o} r_{I \to S}(t) + \frac{p_a R_i}{R_i + p_a R_o} r_{I \to A}(t) + \frac{p_a R_o}{R_i + p_a R_o} r_{A \to I}(t).$$

Incorporating a quarantine of duration $q$ for contacts, the expected post-quarantine transmission

$$R_c(q) = \frac{p_s R_i}{R_i + p_a R_o} R_{I \to S}(q) + \frac{p_a R_i}{R_i + p_a R_o} R_{I \to A}(q) + \frac{p_a R_o}{R_i + p_a R_o} R_{A \to I}(q).$$

***Probability of post-quarantine transmission***

The probability of post-quarantine transmission is specified to be the probability that an infected individual exits quarantine, but can still infect one or more individuals. We calculated this probability under a negative-binomial model appropriate when superspreaders play a role in transmission, as well as a Poisson distribution appropriate when transmission is fairly evenly distributed among infected individuals.

**Negative-binomial distribution**. We specified a negative binomial distribution

$$f(x|k,p) = \frac{\Gamma(k+x)}{\Gamma(k)\Gamma(x+1)} p^k (1-p)^x,$$

with dispersion parameter $k = 0.25$ [3] and $p = \frac{k}{k + R_{q\mapsto}(q)}$, such that the average of the distribution was $R_{q\mapsto}(q)$. Thus, the corresponding probability of post-quarantine transmission with negative binomially-distributed transmissions from a case is

$$P(q) = 1 - f(0|k,p).$$

**Poisson distribution.** Specifying a Poisson distribution producing an expected number of secondary infections post-quarantine transmission of $R_{q\mapsto}(q)$, the probability of transmission after a quarantine of duration $q$ days

$$P(q) = 1 - e^{-R_{q\mapsto}(q)}.$$

***Population prevalence***

Given a cohort size $N$ and a prevalence of $\rho$, the probability of post-quarantine transmission is $1 - (1 - P(q))^{N\rho}$.

***Methods***

**Infectivity function**. We use a Gamma function to specify the infectivity over the disease time course (Supplementary Fig. 1 and Supplementary Fig. 11). We generated the infectivity profile during the pre-symptomatic phase for each duration of the pre-symptomatic period corresponding to each latent period, using the R code provided from He et al [4]. However, as a matter of accounting for the full disease time course, a level of infectivity during the latent period prior to the discrete onset of the distribution provided by He et al [4] must also be specified. Therefore, we specified the infectivity during this early period of infection as $A(e^{m\cdot(t)} - 1)$, where the constants $m$ and $A$ are estimated such that the infectivity function $r(t)$ is smooth and continuous over the entire disease time course. Since the infectivity profile after the latent stage is described by a Gamma function (which has an initial value of zero), we truncate the exponential function at time $t_L + \Delta t$, where $t_L$ is the duration of the latent period; $\Delta t$ was set as the difference between $t_L$ and the upper bound of $t_L$ (where the difference in the log-likelihood at $t_L$ and at $t_L + \Delta t$ was 1.96 [5]).

**Diagnostic sensitivity function**. To characterize the diagnostic sensitivity post-symptom onset, we estimated the coefficients of a logistic regression model

$$\ln\left(\frac{s(t)}{1-s(t)}\right) = \sum_{j=0}^{N} \beta_j (t - t_S)^j,$$

by fitting the function $s(t)$ to diagnostic test-sensitivity data from day zero to 25 days post-symptom onset [6] through the minimization of least squares

$$RSS = \min_{\beta} \sum_{i=0}^{25} \left(\ln\left(\frac{s(i+t_S)}{1-s(i+t_S)}\right) - \ln\left(\frac{\tilde{s}_i}{1-\tilde{s}_i}\right)\right)^2,$$

where $\tilde{s}_i$ denotes the observed diagnostic sensitivity at day $i$ post-symptom onset. The peak infectivity occurs prior to symptom onset from the inferred infectivity curves [4], implying that the infectivity curve is monotonically decreasing over time after symptom onset. To be consistent, the sensitivity should also be monotonically decreasing over time after symptom onset as infectivity (a proxy for the viral load) is decreasing. Therefore, a constraint that the maximum sensitivity after symptom onset occurred at time zero was included in the estimation of the coefficients of the logistic regression model.

To select the number of coefficients in the logistic regression model, we used the Akaike information criterion,

$$\text{AIC} = 2(N + 1) + 26 \ln(RSS),$$

where there are $N + 1$ coefficients being estimated for the 26 data points. The logistic regression model with the lowest AIC value was used to determine the diagnostic sensitivity.

We used diagnostic test-sensitivity data from zero to 25 days post-symptom onset [6] and the infectivity profile post-symptom onset [4] to construct a mapping from infectivity to diagnostic sensitivity, then used that mapping to infer the diagnostic sensitivity during the incubation period from the infectivity pre-symptom onset. To infer the diagnostic sensitivity during the unobserved incubation period, we defined an interpolation function for the diagnostic sensitivity based on the Cartesian pairing of $r(t)$ and $s(t)$ from symptom onset. Since the peak of infectivity occurred

prior to symptom onset, we performed a slight extrapolation of the function $s(t)$ determined by logistic regression. This extrapolation lies within a small range between the symptom-onset diagnostic sensitivity of 0.96 and an upper limit of 1.0 for each latent period considered, so that our results are not sensitive to this extrapolation.

## Supplementary Tables

**Supplementary Table 1:** Parameter descriptions and values used to assess quarantine and testing strategies

| Description | Parameter | Value | Reference |
|---|---|---|---|
| Basic reproductive number | $R_0$ | 2.5 and 2.0 | [7] |
| Basic reproductive number for symptomatic infection | $R_{0,s}$ | $R_0$ | [2] |
| Basic reproductive number for asymptomatic infection | $R_{0,a}$ | $R_0$ | [2] |
| Incubation period | $t_S$ | 8.29 days | [8] |
| Duration of disease in asymptomatic individuals | $t_e$ | $t_S$ + 20 days | [9–11] |
| Proportion of infections that are asymptomatic | $p_a$ | 30.8%<br>22.6% | [12,13]<br>[13,14] |
| Latent period | $t_L$ | 2.9<br>1.9 and 3.9 | [15] |

## Supplementary Figures

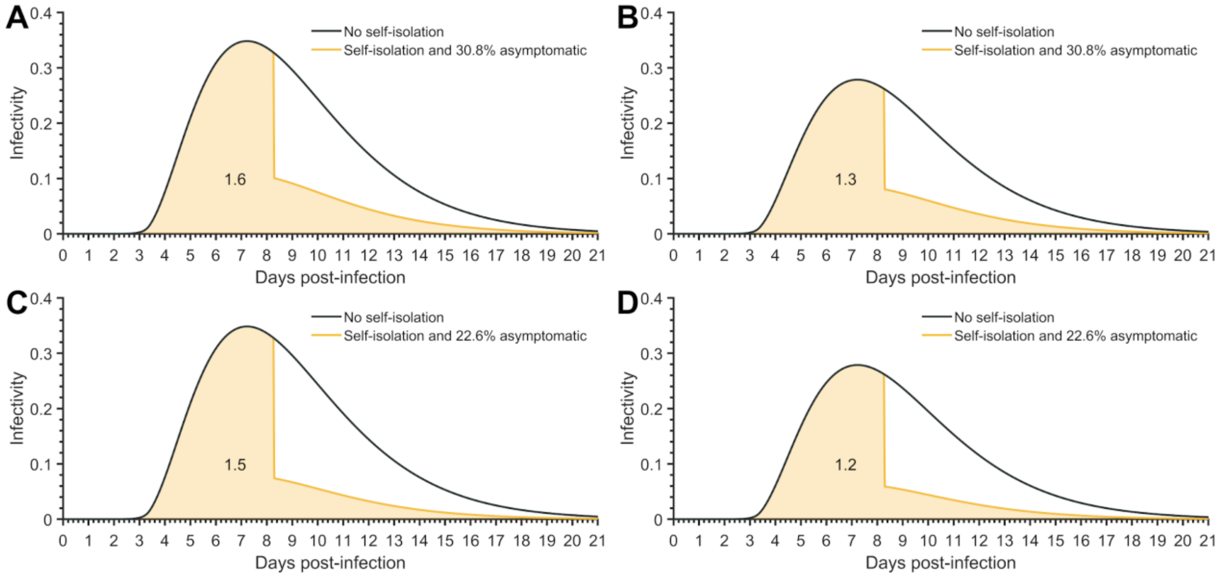


Supplementary Figure 1. **Average infectivity profile for a known time of infection under distinct parameterizations.** Average infectivity profile for a known time of infection under no self-isolation upon symptom onset (black) and perfect isolation upon symptom onset (yellow line) for (A) $R_0$ = 2.5 and 30.8% of infections being asymptomatic (resulting in 1.6 secondary infections, yellow fill), (B) $R_0$ = 2 and 30.8% of infections being asymptomatic (resulting in 1.3 secondary infections, yellow fill), (C) $R_0$ = 2.5 and 22.6% of infections being asymptomatic (resulting in 1.5 secondary infections, yellow fill) and (D) $R_0$ = 2 and 22.6% of infections being asymptomatic (resulting in 1.2 secondary infections, yellow fill).

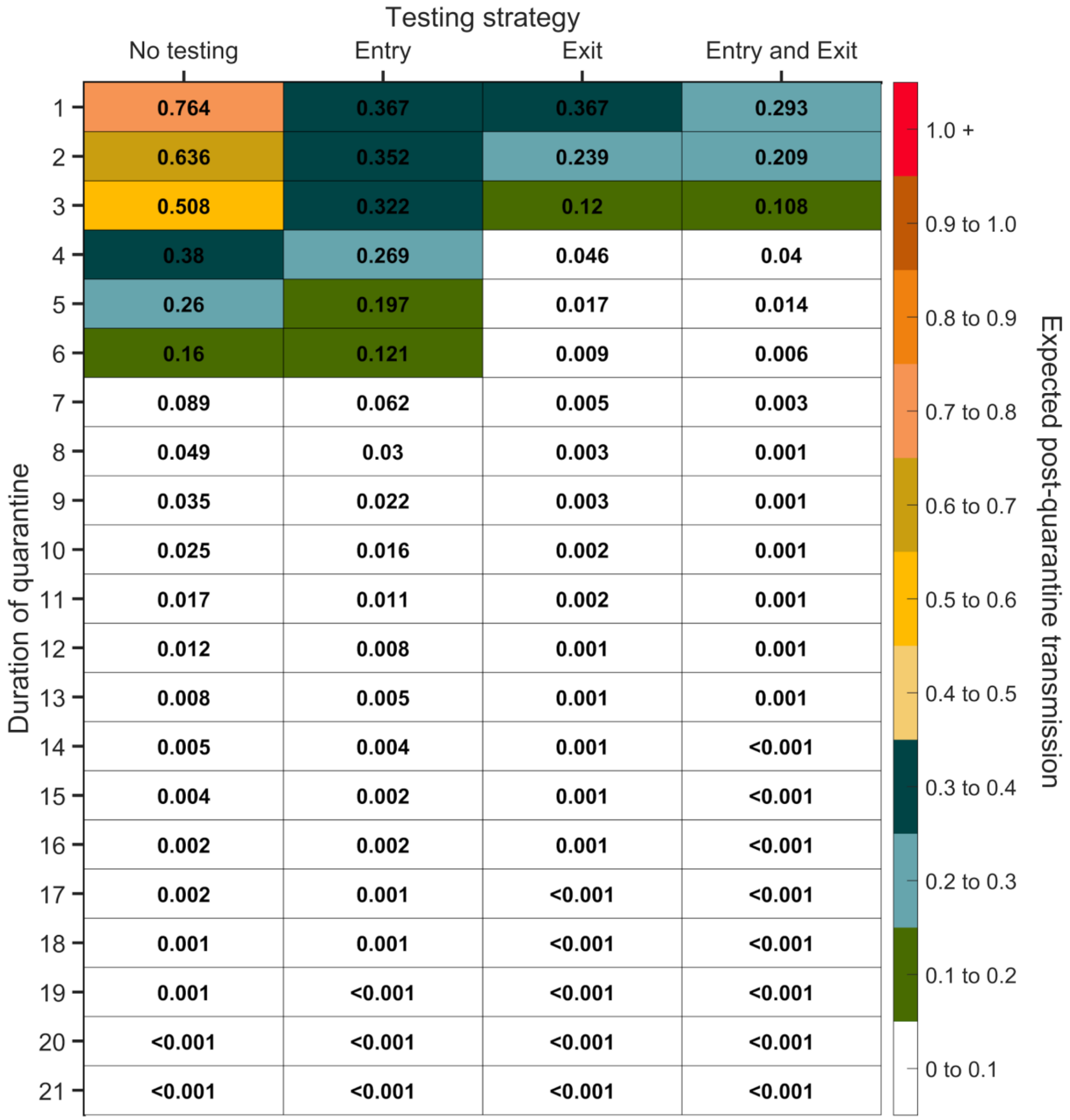


Supplementary Figure 2. **Expected post-quarantine infections for travel quarantine.** Expected post-quarantine infections for durations of quarantine of 1–21 days, with an incubation period of 8.29 days, a latent period of 2.9 days, 30.8% of infections being asymptomatic, perfect self-isolation of symptomatic infections when symptomatic, uniform entry within the incubation period by symptomatic cases, and uniform entry across the disease time course for asymptomatic cases, with no testing, testing on entry, testing on exit, and testing on entry and exit. Because of the time required to obtain test results, sampling for the test on exit was assumed to occur the day before the quarantine was completed.

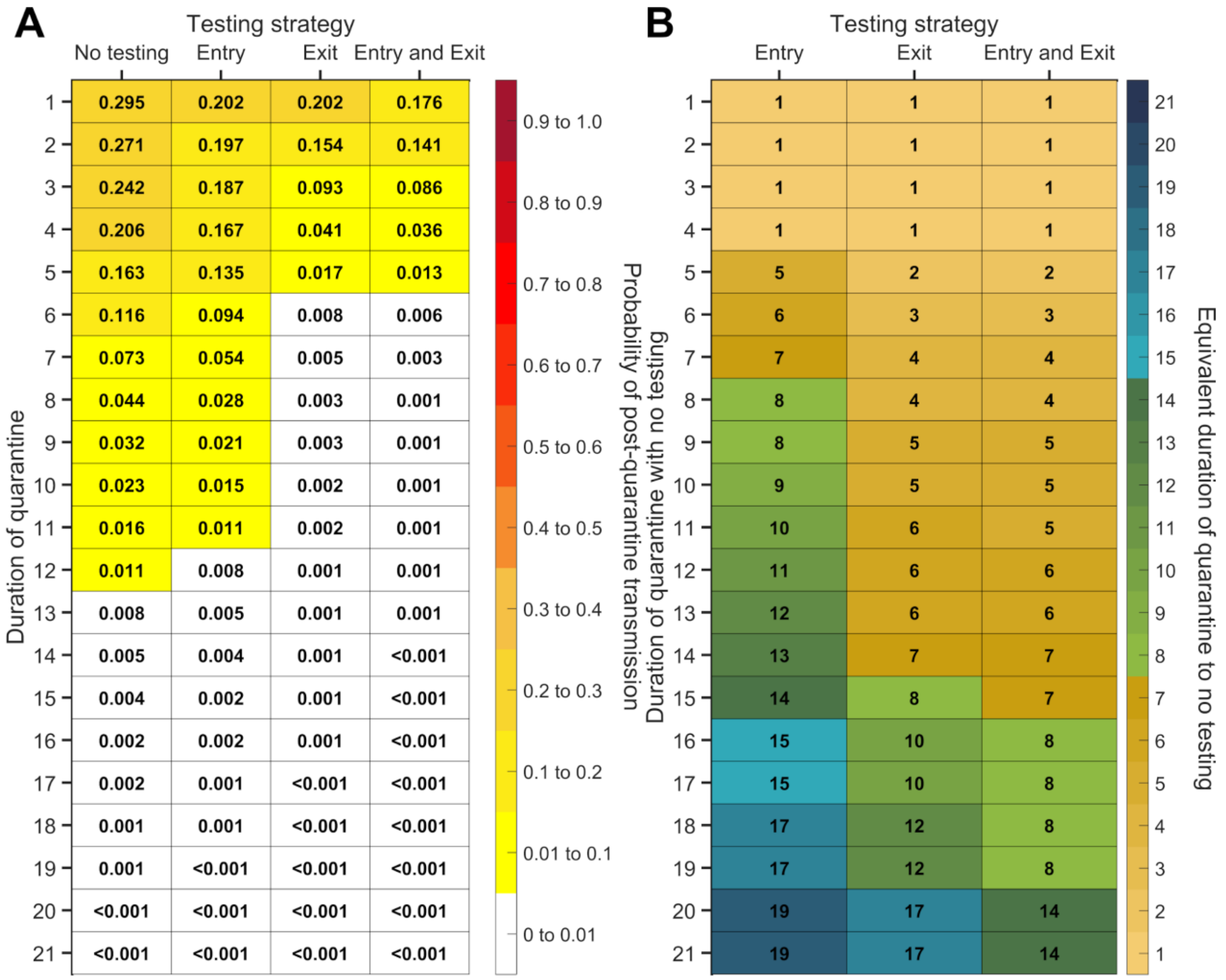


Supplementary Figure 3. **Probability of post-quarantine transmission and equivalent quarantine duration with testing for travel quarantine.** For durations of quarantine from 1–21 days, when a symptomatic individual enters quarantine uniformly within the incubation period and asymptomatic individuals enter uniformly across the disease time course, with an incubation period of 8.29 days, a latent period of 2.9 days, with 30.8% of infections being asymptomatic, and perfect self-isolation of symptomatic infections, (A) the probability of post-quarantine transmission (probability of one or more post-quarantine infections) with no testing, when tested upon entry to quarantine, when tested on exit from quarantine, and when tested on entry and exit from quarantine, and (B) the durations of quarantine with testing on entry, testing on exit, and testing on entry and exit that perform just as well or better than a quarantine with no testing. Cells that share a background color in common indicate equivalent durations of quarantine associated with each of the testing strategies. Because of the time required to obtain test results, sampling for the test on exit is assumed to occur the day before the quarantine is complete.

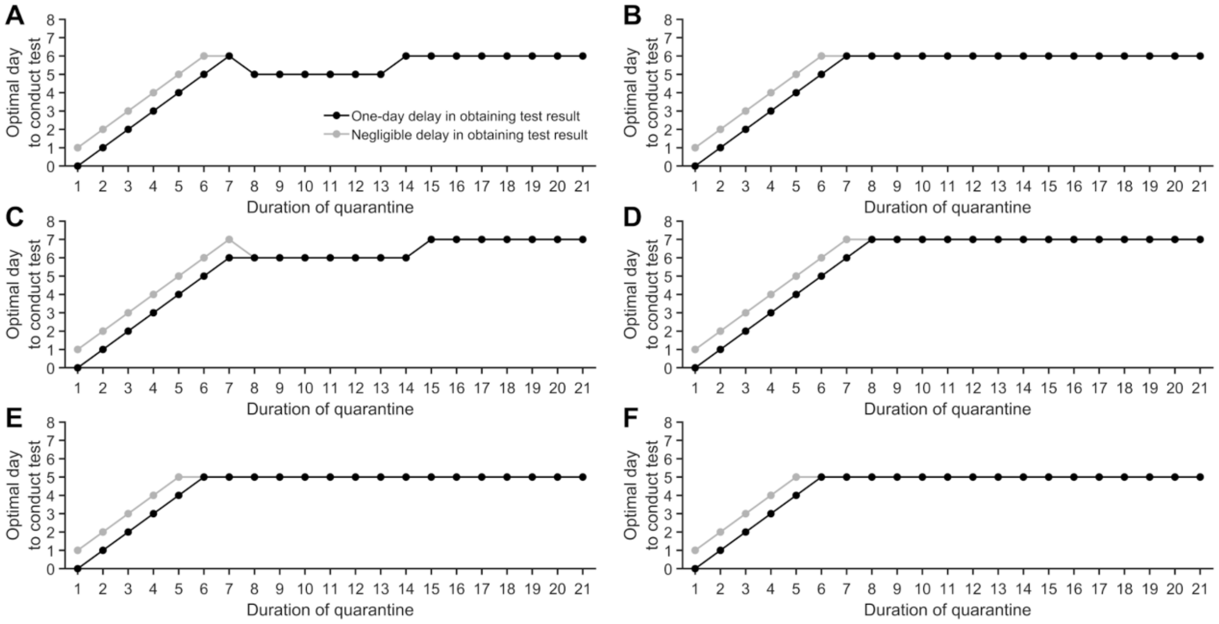


Supplementary Figure 4. **Optimal testing day for travel quarantine and for quarantine of traced contacts.** With 30.8% of infections asymptomatic, perfect self-isolation of symptomatic infections, and an incubation period of 8.29 days, the optimal day of testing to obtain the minimum post-quarantine transmission specifying a latent period of (A) 2.9 days with uniform entry into quarantine, (B) 2.9 days and entry into quarantine as a traced contact, (C) 1.9 days and uniform entry into quarantine, (D) 1.9 days and entry into quarantine as a traced contact, (E) 3.9 days and uniform entry into quarantine, and (F) 3.9 days and entry into quarantine as a traced contact for a one-day delay in obtaining test results (black) and a negligible delay in obtaining test results (gray).

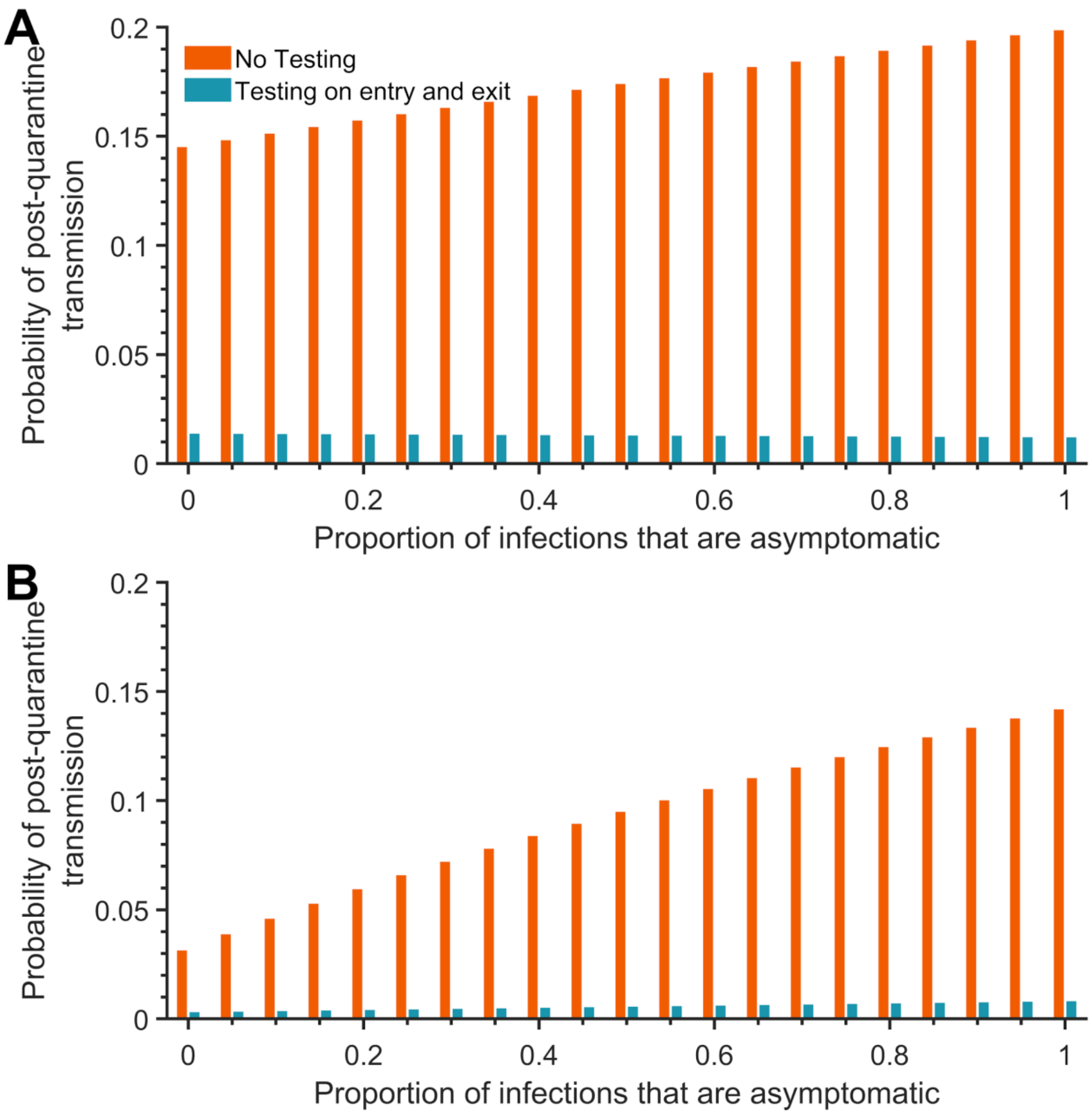


Supplementary Figure 5. **Probability of post-quarantine transmission dependent on the proportion of infections that are asymptomatic.** With perfect self-isolation of symptomatic infections, an incubation period of 8.29 days and a latent period of 2.9 days, and proportions from 0–1 of infections being asymptomatic, the probability of post-quarantine transmission (probability of one or more post-quarantine infections) when symptomatic individuals enter quarantine uniformly within the incubation period and asymptomatic individuals enter uniformly across the disease time course, with no testing (red) and when tested on entry and exit from quarantine (blue) for a (A) five-day quarantine and a (B) seven-day quarantine. The exit test was assumed to occur 96 h after entry into quarantine.

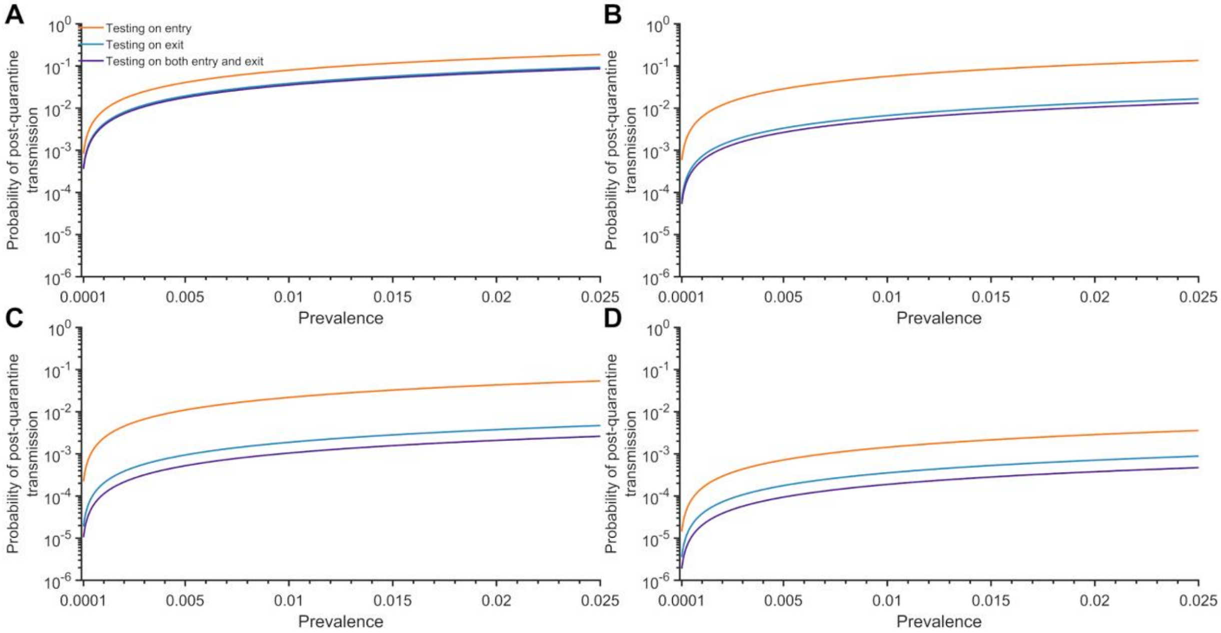


Supplementary Figure 6. **Probability of post-quarantine transmission for a cohort, dependent on community prevalence.** Specifying an incubation period of 8.29 days and a latent period of 2.9 days, the probability of anu post-quarantine transmission accounting for underlying community prevalence in a cohort (crew) of 40 employees for testing on entry (orange), testing on exit (blue), and testing on both entry and exit (purple) for a (A) three-day quarantine, (B) five-day quarantine, (C) seven-day quarantine, and (D) 14-day quarantine. Because of the time required to obtain test results, sampling for the test on exit is assumed to occur the day before the quarantine is complete.

Testing strategy

| Duration of quarantine | No testing | Entry | Exit | Entry and Exit |
|---|---|---|---|---|
| 1 | 1.15 | 0.722 | 0.722 | 0.612 |
| 2 | 1.058 | 0.707 | 0.494 | 0.446 |
| 3 | 0.937 | 0.666 | 0.253 | 0.232 |
| 4 | 0.774 | 0.583 | 0.099 | 0.087 |
| 5 | 0.586 | 0.461 | 0.04 | 0.031 |
| 6 | 0.407 | 0.324 | 0.022 | 0.016 |
| 7 | 0.267 | 0.208 | 0.014 | 0.009 |
| 8 | 0.177 | 0.136 | 0.01 | 0.006 |
| 9 | 0.129 | 0.1 | 0.008 | 0.005 |
| 10 | 0.092 | 0.073 | 0.007 | 0.004 |
| 11 | 0.065 | 0.052 | 0.006 | 0.004 |
| 12 | 0.045 | 0.036 | 0.005 | 0.003 |
| 13 | 0.031 | 0.025 | 0.004 | 0.003 |
| 14 | 0.021 | 0.017 | 0.003 | 0.002 |
| 15 | 0.014 | 0.011 | 0.002 | 0.002 |
| 16 | 0.009 | 0.007 | 0.002 | 0.001 |
| 17 | 0.006 | 0.005 | 0.001 | 0.001 |
| 18 | 0.004 | 0.003 | 0.001 | 0.001 |
| 19 | 0.003 | 0.002 | 0.001 | 0.001 |
| 20 | 0.002 | 0.001 | 0.001 | <0.001 |
| 21 | 0.001 | 0.001 | <0.001 | <0.001 |

Expected post-quarantine transmission: 1.0 +; 0.9 to 1.0; 0.8 to 0.9; 0.7 to 0.8; 0.6 to 0.7; 0.5 to 0.6; 0.4 to 0.5; 0.3 to 0.4; 0.2 to 0.3; 0.1 to 0.2; 0 to 0.1

Supplementary Figure 7. **Expected post-quarantine infections for traced contacts.** Expected post-quarantine infections for durations of quarantine of 1–21 days, with an incubation period of 8.29 days, a latent period of 2.9 days, 30.8% of infections being asymptomatic, perfect self-isolation of symptomatic infections when symptomatic, and entry through contact tracing, with no testing, testing on entry, testing on exit, and testing on entry and exit. Because of the time required to obtain test results, sampling for the test on exit was assumed to occur the day before the quarantine was completed.

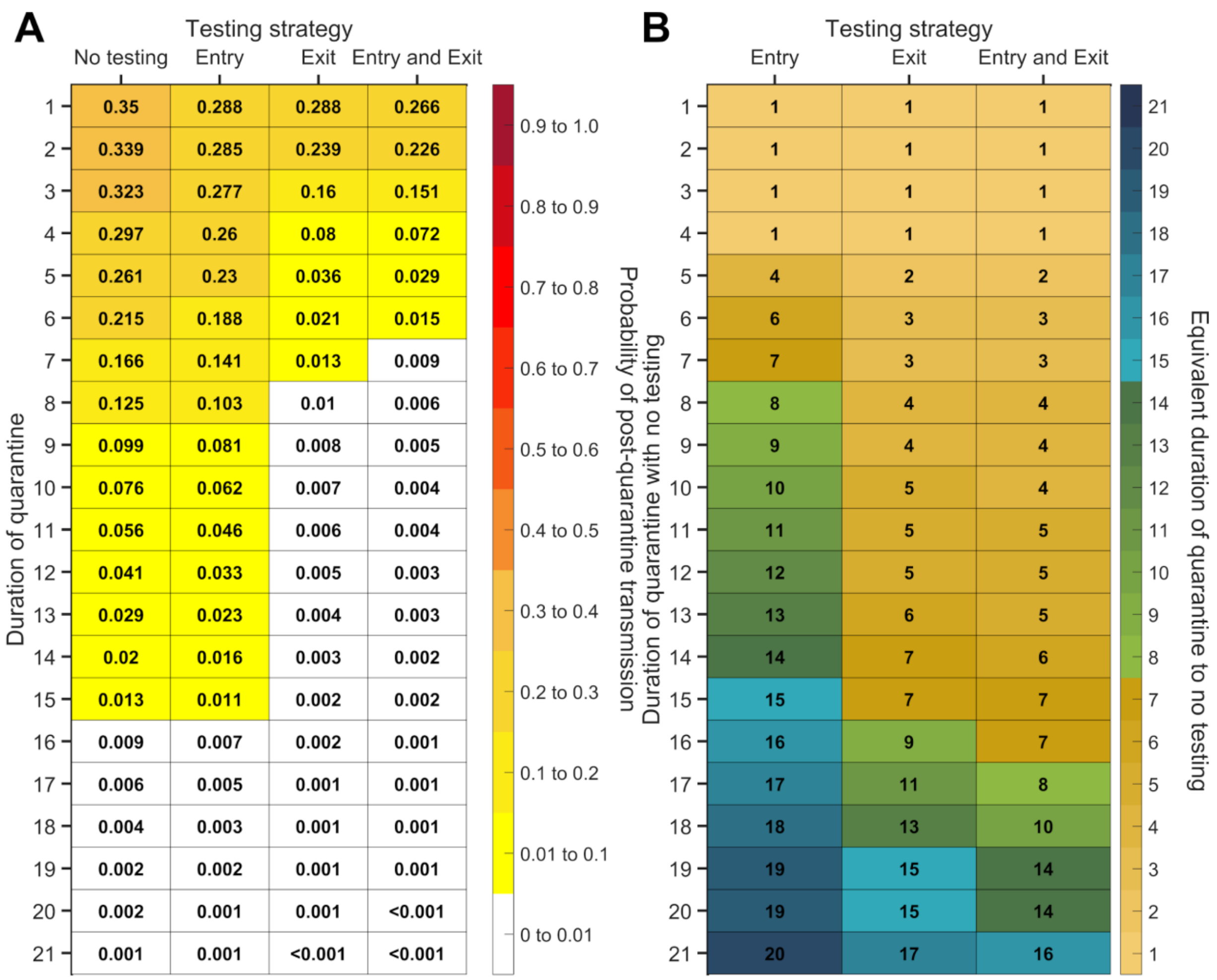


Supplementary Figure 8. **Probability of post-quarantine transmission and equivalent quarantine duration with testing for traced contacts.** For durations of quarantine from 1–21 days, when an individual enters quarantine through contact tracing, with an incubation period of 8.29 days, a latent period of 2.9 days, with 30.8% of infections being asymptomatic, and perfect self-isolation of symptomatic infections, (A) the probability of post-quarantine transmission (probability of one or more post-quarantine infections) with no testing, when tested upon entry to quarantine, when tested on exit from quarantine, and when tested on entry and exit from quarantine, and (B) the durations of quarantine with testing on entry, testing on exit, and testing on entry and exit that perform just as well or better than a quarantine with no testing. Cells that share a background color in common indicate equivalent durations of quarantine associated with each of the testing strategies. Because of the time required to obtain test results, sampling for the test on exit is assumed to occur the day before the quarantine is complete.

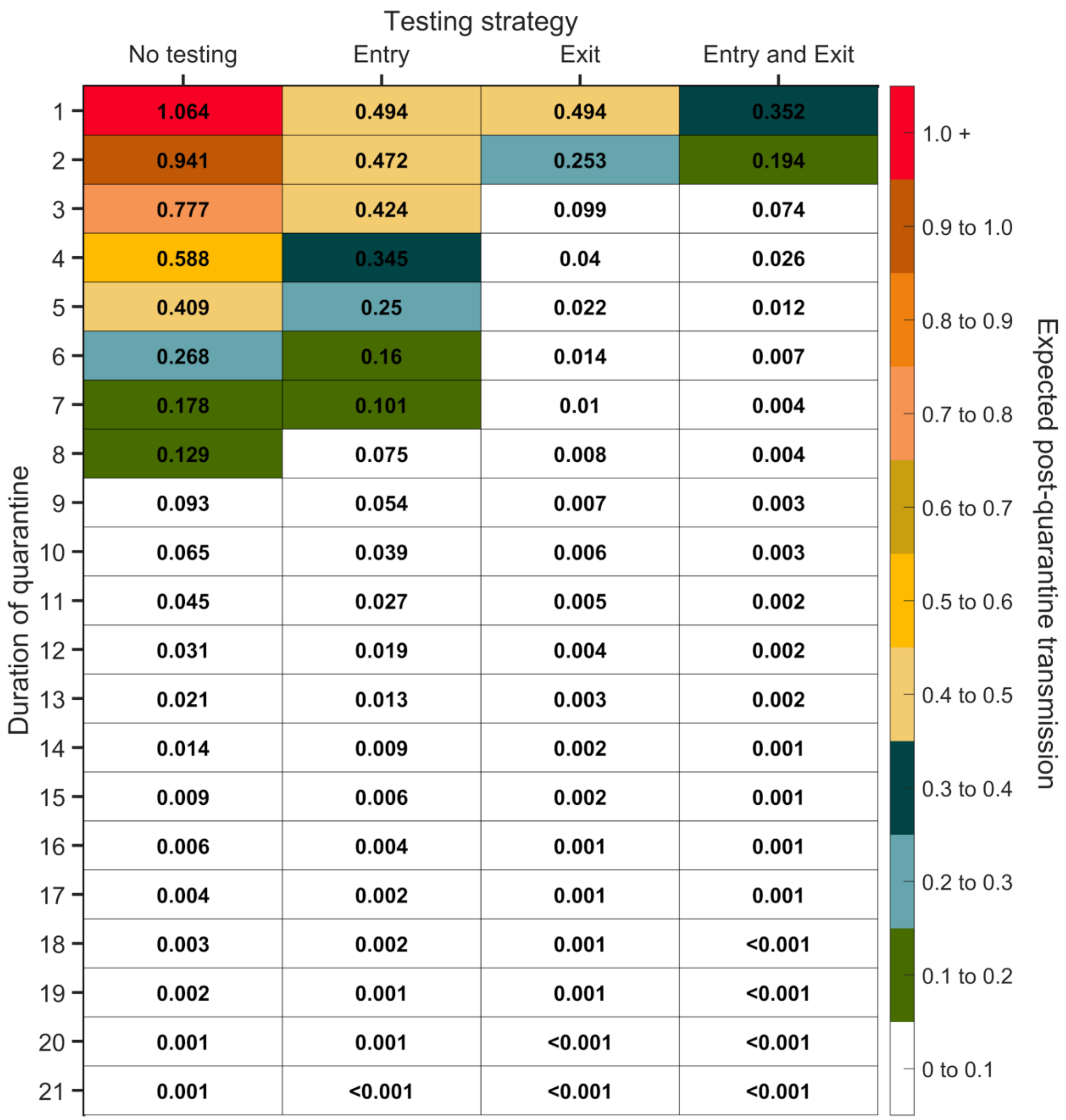


Supplementary Figure 9. **Expected post-quarantine infections for traced contacts with a one-day tracing delay.** Expected post-quarantine infections for durations of quarantine of 1–21 days, with an incubation period of 8.29 days, a latent period of 2.9 days, 30.8% of infections being asymptomatic, perfect self-isolation of symptomatic infections when symptomatic, and entry through contact tracing with a one-day tracing delay, with no testing, testing on entry, testing on exit, and testing on entry and exit. Because of the time required to obtain test results, sampling for the test on exit was assumed to occur the day before the quarantine was completed.

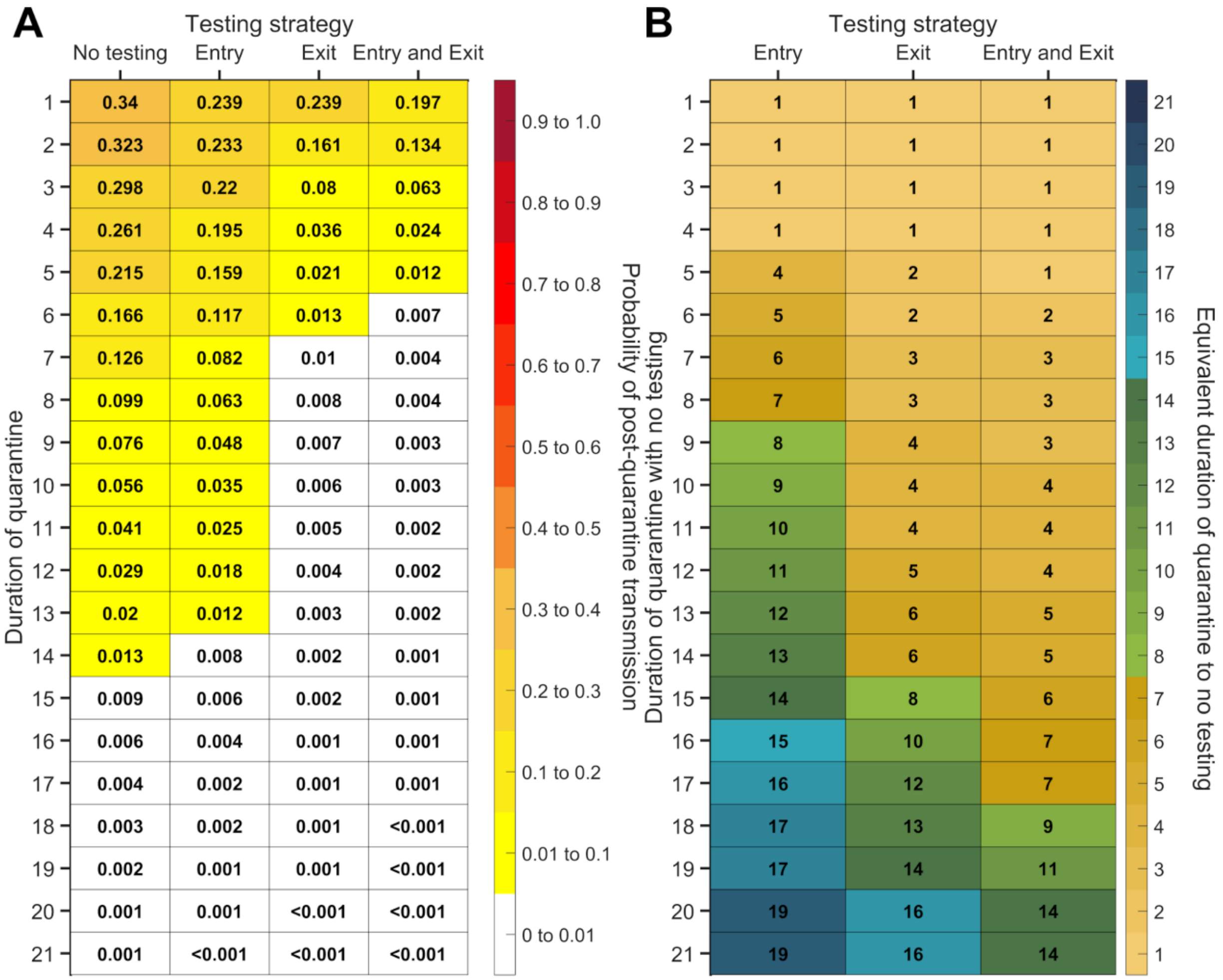


| Duration of quarantine | No testing | Entry | Exit | Entry and Exit |
|---|---|---|---|---|
| 1 | 0.34 | 0.239 | 0.239 | 0.197 |
| 2 | 0.323 | 0.233 | 0.161 | 0.134 |
| 3 | 0.298 | 0.22 | 0.08 | 0.063 |
| 4 | 0.261 | 0.195 | 0.036 | 0.024 |
| 5 | 0.215 | 0.159 | 0.021 | 0.012 |
| 6 | 0.166 | 0.117 | 0.013 | 0.007 |
| 7 | 0.126 | 0.082 | 0.01 | 0.004 |
| 8 | 0.099 | 0.063 | 0.008 | 0.004 |
| 9 | 0.076 | 0.048 | 0.007 | 0.003 |
| 10 | 0.056 | 0.035 | 0.006 | 0.003 |
| 11 | 0.041 | 0.025 | 0.005 | 0.002 |
| 12 | 0.029 | 0.018 | 0.004 | 0.002 |
| 13 | 0.02 | 0.012 | 0.003 | 0.002 |
| 14 | 0.013 | 0.008 | 0.002 | 0.001 |
| 15 | 0.009 | 0.006 | 0.002 | 0.001 |
| 16 | 0.006 | 0.004 | 0.001 | 0.001 |
| 17 | 0.004 | 0.002 | 0.001 | 0.001 |
| 18 | 0.003 | 0.002 | 0.001 | <0.001 |
| 19 | 0.002 | 0.001 | 0.001 | <0.001 |
| 20 | 0.001 | 0.001 | <0.001 | <0.001 |
| 21 | 0.001 | <0.001 | <0.001 | <0.001 |

| Duration of quarantine with no testing | Entry | Exit | Entry and Exit |
|---|---|---|---|
| 1 | 1 | 1 | 1 |
| 2 | 1 | 1 | 1 |
| 3 | 1 | 1 | 1 |
| 4 | 1 | 1 | 1 |
| 5 | 4 | 2 | 1 |
| 6 | 5 | 2 | 2 |
| 7 | 6 | 3 | 3 |
| 8 | 7 | 3 | 3 |
| 9 | 8 | 4 | 3 |
| 10 | 9 | 4 | 4 |
| 11 | 10 | 4 | 4 |
| 12 | 11 | 5 | 4 |
| 13 | 12 | 6 | 5 |
| 14 | 13 | 6 | 5 |
| 15 | 14 | 8 | 6 |
| 16 | 15 | 10 | 7 |
| 17 | 16 | 12 | 7 |
| 18 | 17 | 13 | 9 |
| 19 | 17 | 14 | 11 |
| 20 | 19 | 16 | 14 |
| 21 | 19 | 16 | 14 |

Supplementary Figure 10. **Probability of post-quarantine transmission and equivalent quarantine duration with testing for traced contacts with a one-day tracing delay.** For durations of quarantine from 1–21 days, when an individual enters quarantine through contact tracing with a one-day tracing delay, with an incubation period of 8.29 days, a latent period of 2.9 days, with 30.8% of infections being asymptomatic, and perfect self-isolation of symptomatic infections, (A) the probability of post-quarantine transmission (probability of one or more post-quarantine infections) with no testing, when tested upon entry to quarantine, when tested on exit from quarantine, and when tested on entry and exit from quarantine, and (B) the durations of quarantine with testing on entry, testing on exit, and testing on entry and exit that perform just as well or better than a quarantine with no testing. Cells that share a background color in common indicate equivalent durations of quarantine associated with each of the testing strategies. Because of the time required to obtain test results, sampling for the test on exit is assumed to occur the day before the quarantine is complete.

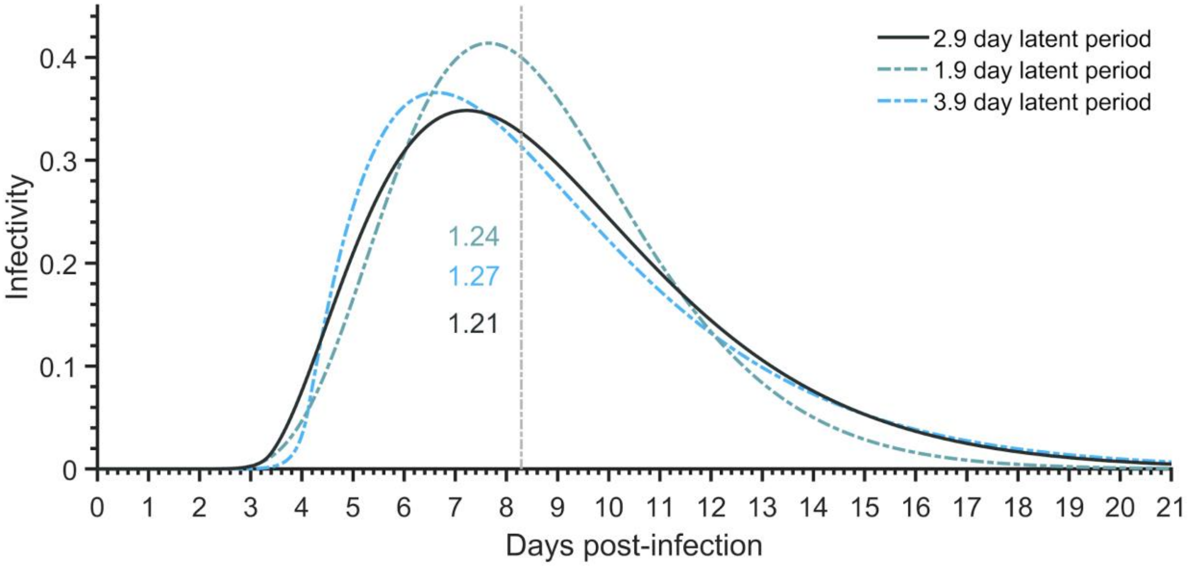


Supplementary Figure 11. **Infectivity profiles corresponding to latent periods of 1.9, 2.9, and 3.9 days.** Infectivity profiles of an individual for an incubation period of 8.29 days and assuming no self-isolation upon symptom onset, corresponding to the reported duration of the latent period (2.9, black), and corresponding to latent periods one day longer (3.9, dashed blue), and one day shorter (1.9, dashed green), and numbers of secondary infections that occur within the incubation period for a 2.9-day latent period (1.21, black), for a 3.9-day latent period (1.27, blue), and for a 1.9-day latent period (1.24, green).

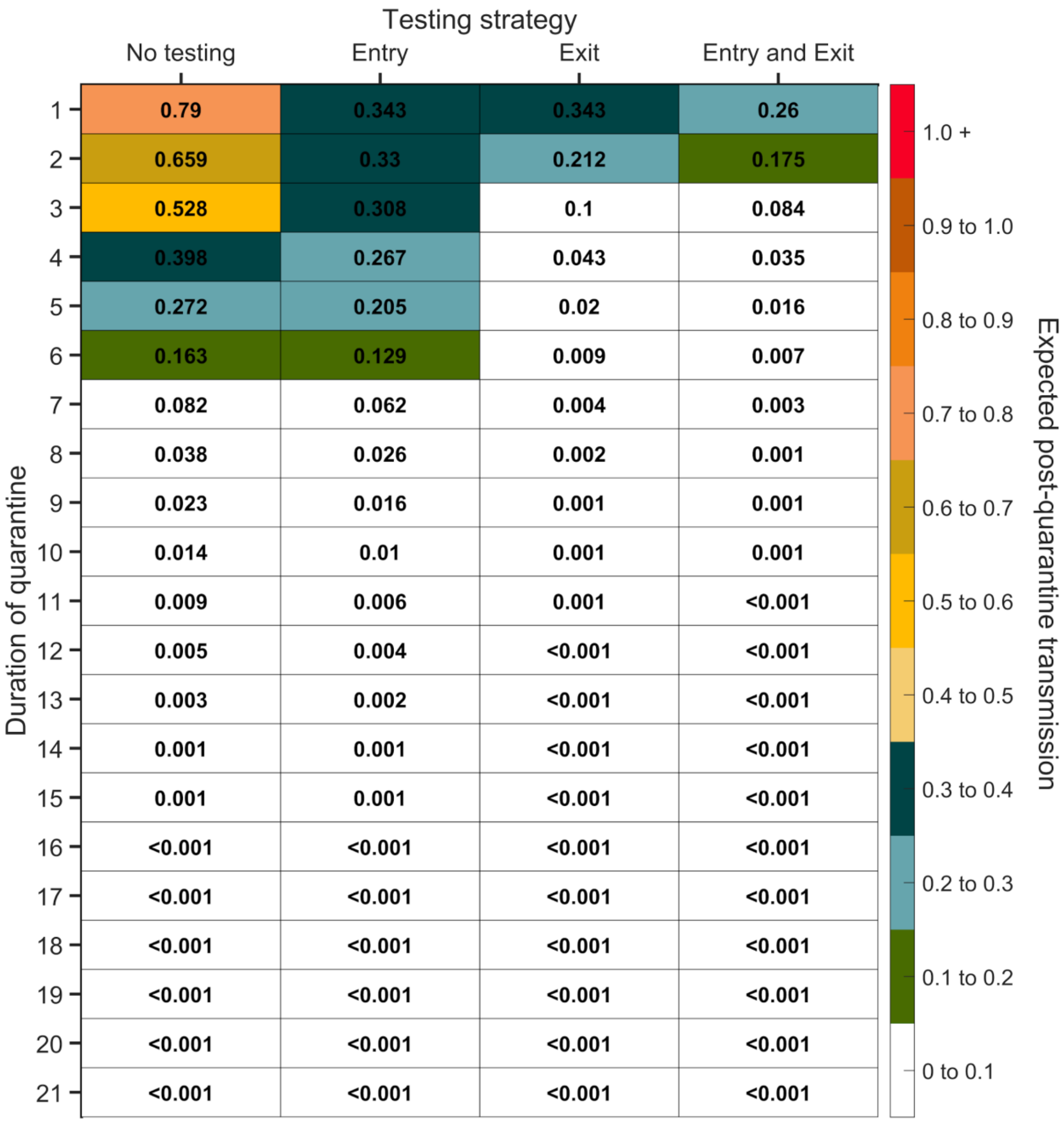


Supplementary Figure 12. **Expected post-quarantine infections for travel quarantine, specifying a 1.9-day latent period.** Expected post-quarantine infections for durations of quarantine of 1–21 days, with an incubation period of 8.29 days, 30.8% of infections being asymptomatic, perfect self-isolation of symptomatic infections when symptomatic, uniform entry within the incubation period by symptomatic cases, and uniform entry across the disease time course for asymptomatic cases, with no testing, testing on entry, testing on exit, and testing on entry and exit. Because of the time required to obtain test results, sampling for the test on exit was assumed to occur the day before the quarantine was completed.

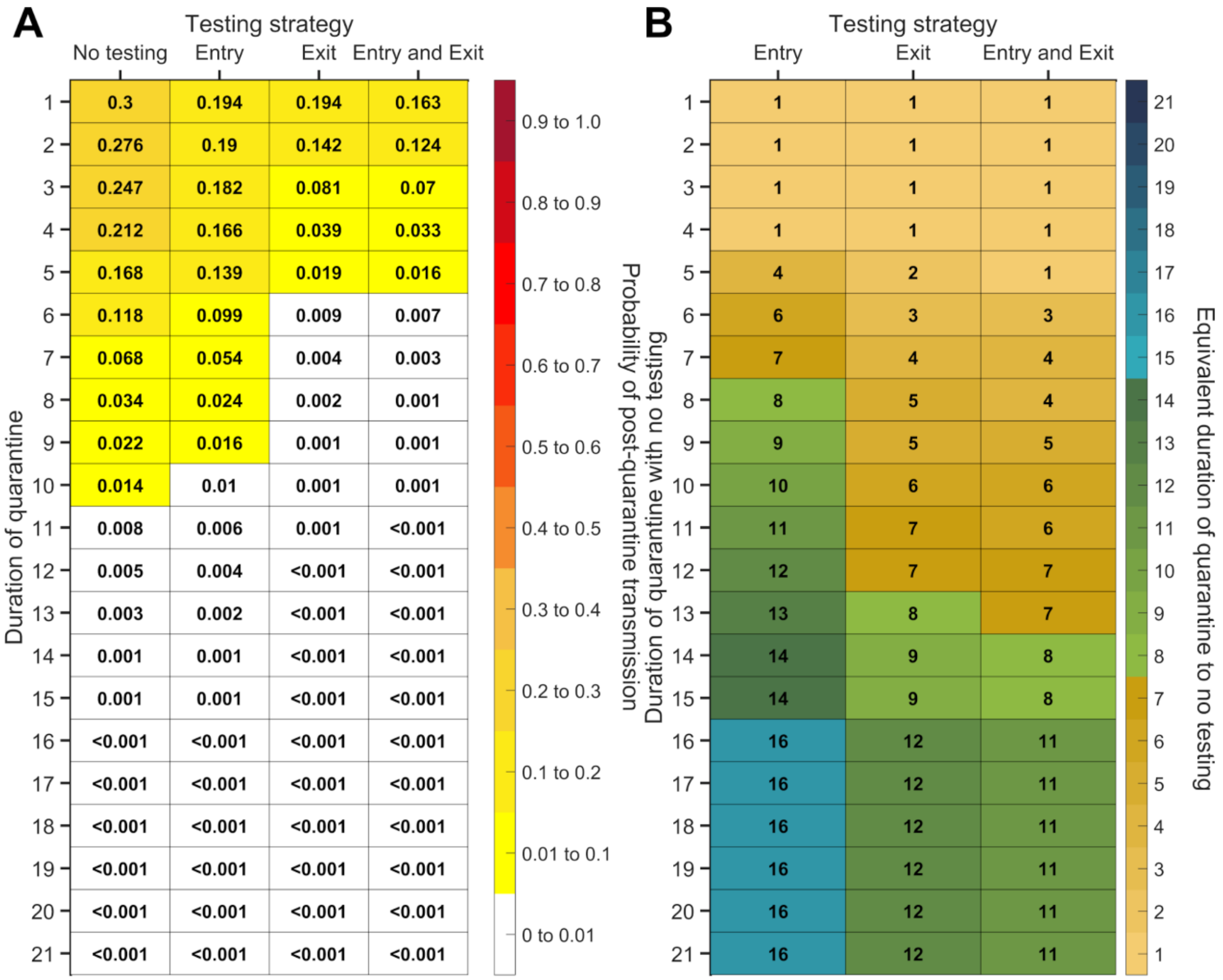


| Duration of quarantine | No testing | Entry | Exit | Entry and Exit |
|---|---|---|---|---|
| 1 | 0.3 | 0.194 | 0.194 | 0.163 |
| 2 | 0.276 | 0.19 | 0.142 | 0.124 |
| 3 | 0.247 | 0.182 | 0.081 | 0.07 |
| 4 | 0.212 | 0.166 | 0.039 | 0.033 |
| 5 | 0.168 | 0.139 | 0.019 | 0.016 |
| 6 | 0.118 | 0.099 | 0.009 | 0.007 |
| 7 | 0.068 | 0.054 | 0.004 | 0.003 |
| 8 | 0.034 | 0.024 | 0.002 | 0.001 |
| 9 | 0.022 | 0.016 | 0.001 | 0.001 |
| 10 | 0.014 | 0.01 | 0.001 | 0.001 |
| 11 | 0.008 | 0.006 | 0.001 | <0.001 |
| 12 | 0.005 | 0.004 | <0.001 | <0.001 |
| 13 | 0.003 | 0.002 | <0.001 | <0.001 |
| 14 | 0.001 | 0.001 | <0.001 | <0.001 |
| 15 | 0.001 | 0.001 | <0.001 | <0.001 |
| 16 | <0.001 | <0.001 | <0.001 | <0.001 |
| 17 | <0.001 | <0.001 | <0.001 | <0.001 |
| 18 | <0.001 | <0.001 | <0.001 | <0.001 |
| 19 | <0.001 | <0.001 | <0.001 | <0.001 |
| 20 | <0.001 | <0.001 | <0.001 | <0.001 |
| 21 | <0.001 | <0.001 | <0.001 | <0.001 |

| Duration of quarantine with no testing | Entry | Exit | Entry and Exit |
|---|---|---|---|
| 1 | 1 | 1 | 1 |
| 2 | 1 | 1 | 1 |
| 3 | 1 | 1 | 1 |
| 4 | 1 | 1 | 1 |
| 5 | 4 | 2 | 1 |
| 6 | 6 | 3 | 3 |
| 7 | 7 | 4 | 4 |
| 8 | 8 | 5 | 4 |
| 9 | 9 | 5 | 5 |
| 10 | 10 | 6 | 6 |
| 11 | 11 | 7 | 6 |
| 12 | 12 | 7 | 7 |
| 13 | 13 | 8 | 7 |
| 14 | 14 | 9 | 8 |
| 15 | 14 | 9 | 8 |
| 16 | 16 | 12 | 11 |
| 17 | 16 | 12 | 11 |
| 18 | 16 | 12 | 11 |
| 19 | 16 | 12 | 11 |
| 20 | 16 | 12 | 11 |
| 21 | 16 | 12 | 11 |

Supplementary Figure 13. **Probability of post-quarantine transmission and equivalent quarantine duration with testing for travel quarantine, specifying a latent period of 1.9 days.** For durations of quarantine from 1–21 days, when a symptomatic individual enters quarantine uniformly within the incubation period and asymptomatic individuals enter uniformly across the disease time course, with an incubation period of 8.29 days, with 30.8% of infections being asymptomatic, and perfect self-isolation of symptomatic infections, (A) the probability of post-quarantine transmission (probability of one or more post-quarantine infections) with no testing, when tested upon entry to quarantine, when tested on exit from quarantine, and when tested on entry and exit from quarantine, and (B) the durations of quarantine with testing on entry, testing on exit, and testing on entry and exit that perform just as well or better than a quarantine with no testing. Cells that share a background color in common indicate equivalent durations of quarantine associated with each of the testing strategies. Because of the time required to obtain test results, sampling for the test on exit is assumed to occur the day before the quarantine is complete.

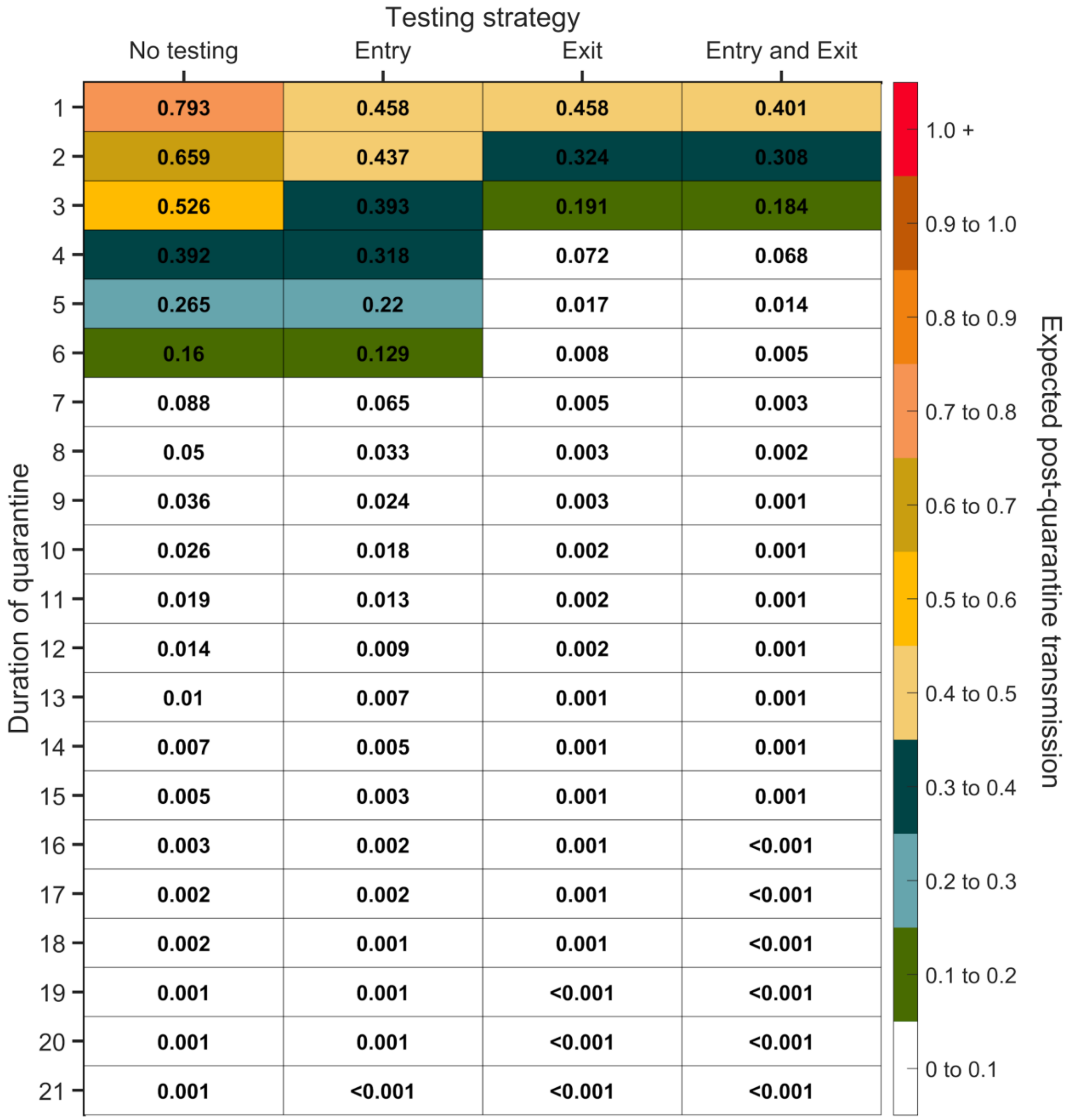


Supplementary Figure 14. **Expected post-quarantine infections for travel quarantine, specifying a 3.9-day latent period.** Expected post-quarantine infections for durations of quarantine of 1–21 days, with an incubation period of 8.29 days, 30.8% of infections being asymptomatic, perfect self-isolation of symptomatic infections when symptomatic, uniform entry within the incubation period by symptomatic cases, and uniform entry across the disease time course for asymptomatic cases, with no testing, testing on entry, testing on exit, and testing on entry and exit. Because of the time required to obtain test results, sampling for the test on exit was assumed to occur the day before the quarantine was completed.

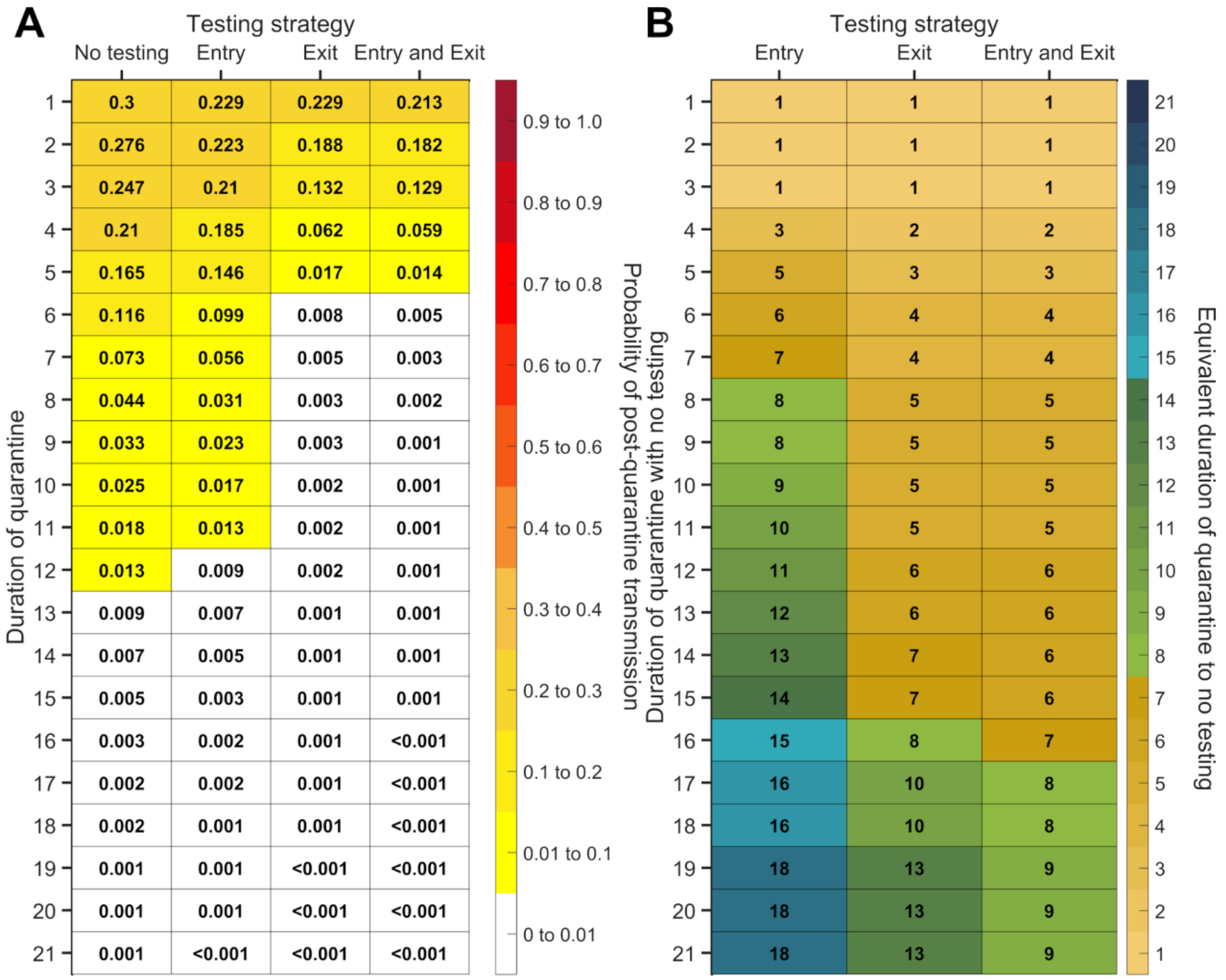


Supplementary Figure 15. **Probability of post-quarantine transmission and equivalent quarantine duration with testing for travel quarantine, specifying a latent period of 3.9 days.** For durations of quarantine from 1–21 days, when a symptomatic individual enters quarantine uniformly within the incubation period and asymptomatic individuals enter uniformly across the disease time course, with an incubation period of 8.29 days, with 30.8% of infections being asymptomatic, and perfect self-isolation of symptomatic infections, (A) the probability of post-quarantine transmission (probability of one or more post-quarantine infections) with no testing, when tested upon entry to quarantine, when tested on exit from quarantine, and when tested on entry and exit from quarantine, and (B) the durations of quarantine with testing on entry, testing on exit, and testing on entry and exit that perform just as well or better than a quarantine with no testing. Cells that share a background color in common indicate equivalent durations of quarantine associated with each of the testing strategies. Because of the time required to obtain test results, sampling for the test on exit is assumed to occur the day before the quarantine is complete.

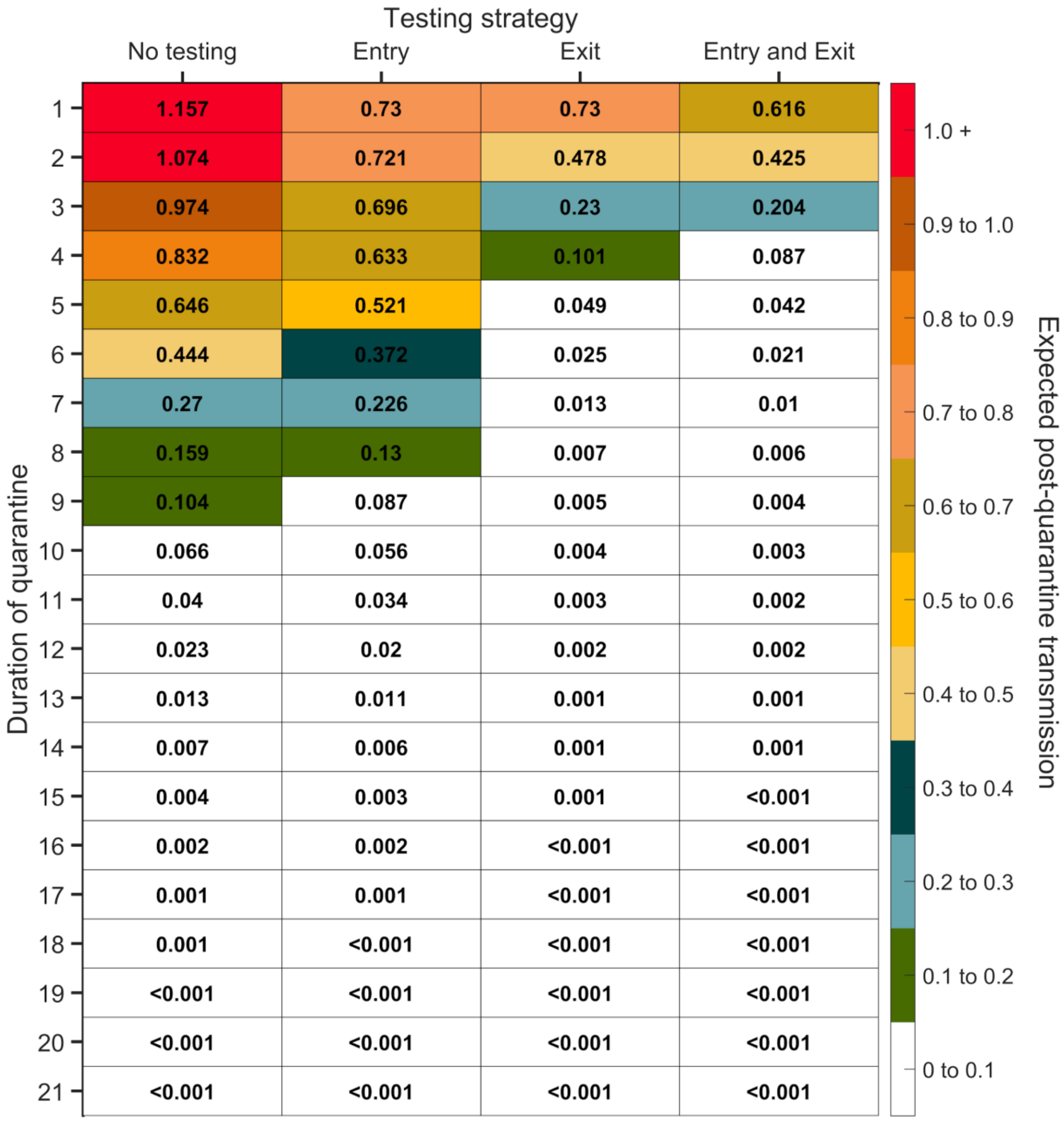


Supplementary Figure 16. **Expected post-quarantine infections for traced contacts, specifying a 1.9-day latent period.** Expected post-quarantine infections for durations of quarantine of 1–21 days, with an incubation period of 8.29 days, 30.8% of infections being asymptomatic, perfect self-isolation of symptomatic infections when symptomatic, and entry through contact tracing, with no testing, testing on entry, testing on exit, and testing on entry and exit. Because of the time required to obtain test results, sampling for the test on exit was assumed to occur the day before the quarantine was completed.

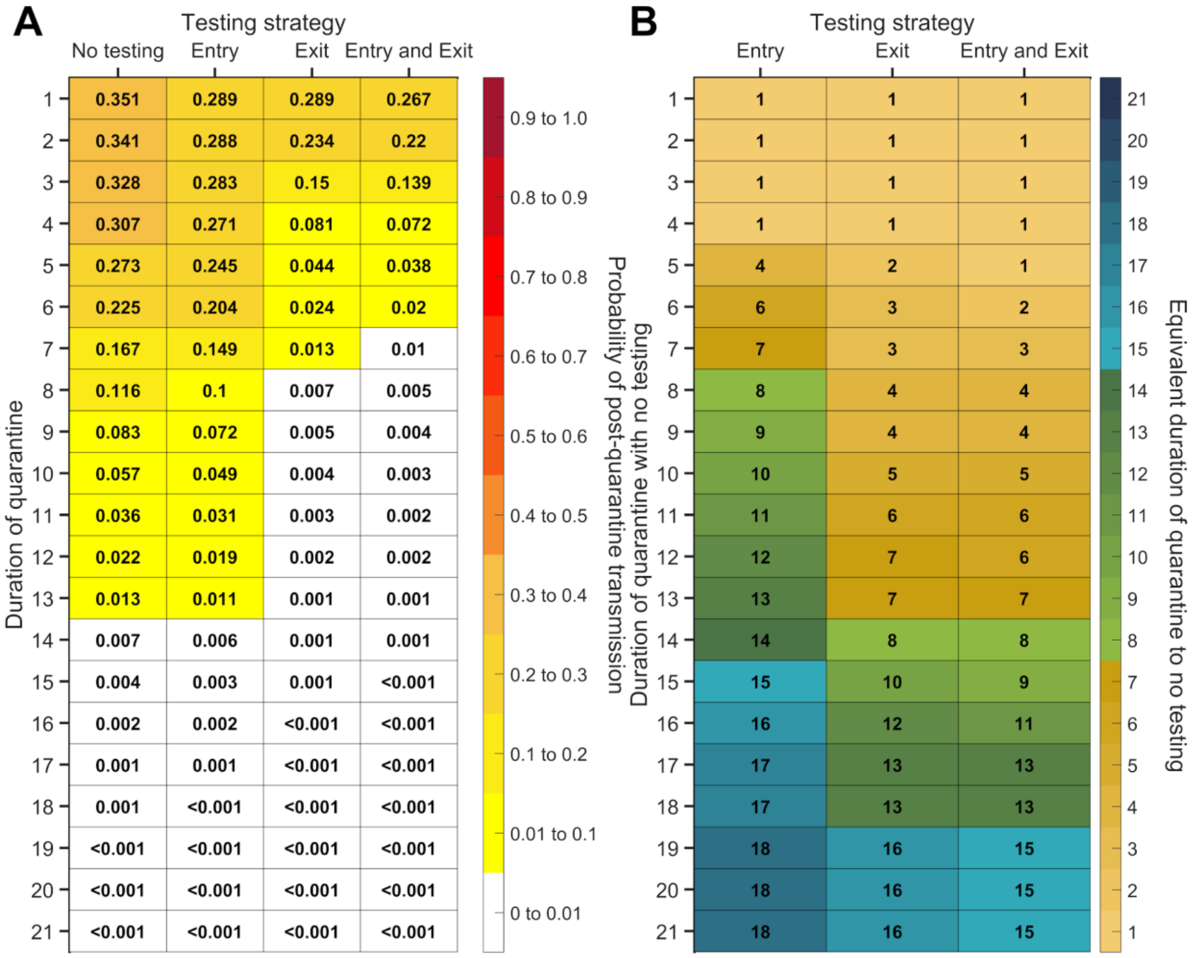


Supplementary Figure 17. **Probability of post-quarantine transmission and equivalent quarantine duration with testing for traced contacts, specifying a latent period of 1.9 days.** For durations of quarantine from 1–21 days, when an individual enters quarantine through contact tracing, with an incubation period of 8.29 days, with 30.8% of infections being asymptomatic, and perfect self-isolation of symptomatic infections, (A) the probability of post-quarantine transmission (probability of one or more post-quarantine infections) with no testing, when tested upon entry to quarantine, when tested on exit from quarantine, and when tested on entry and exit from quarantine, and (B) the durations of quarantine with testing on entry, testing on exit, and testing on entry and exit that perform just as well or better than a quarantine with no testing. Cells that share a background color in common indicate equivalent durations of quarantine associated with each of the testing strategies. Because of the time required to obtain test results, sampling for the test on exit is assumed to occur the day before the quarantine is complete.

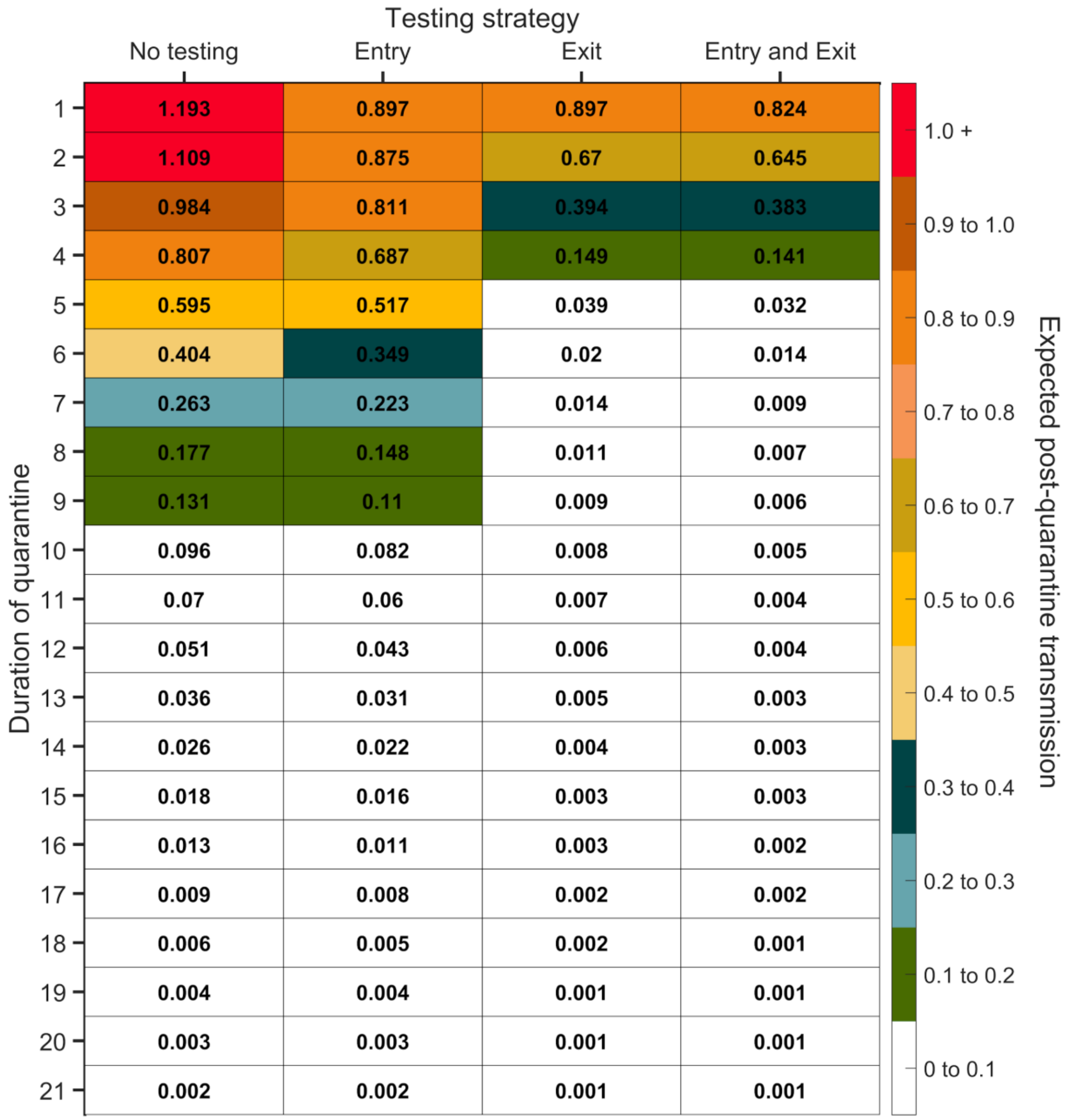


Supplementary Figure 18. **Expected post-quarantine infections for traced contacts, specifying a 3.9-day latent period.** Expected post-quarantine infections for durations of quarantine of 1–21 days, with an incubation period of 8.29 days, 30.8% of infections being asymptomatic, perfect self-isolation of symptomatic infections when symptomatic, and entry through contact tracing, with no testing, testing on entry, testing on exit, and testing on entry and exit. Because of the time required to obtain test results, sampling for the test on exit was assumed to occur the day before the quarantine was completed.

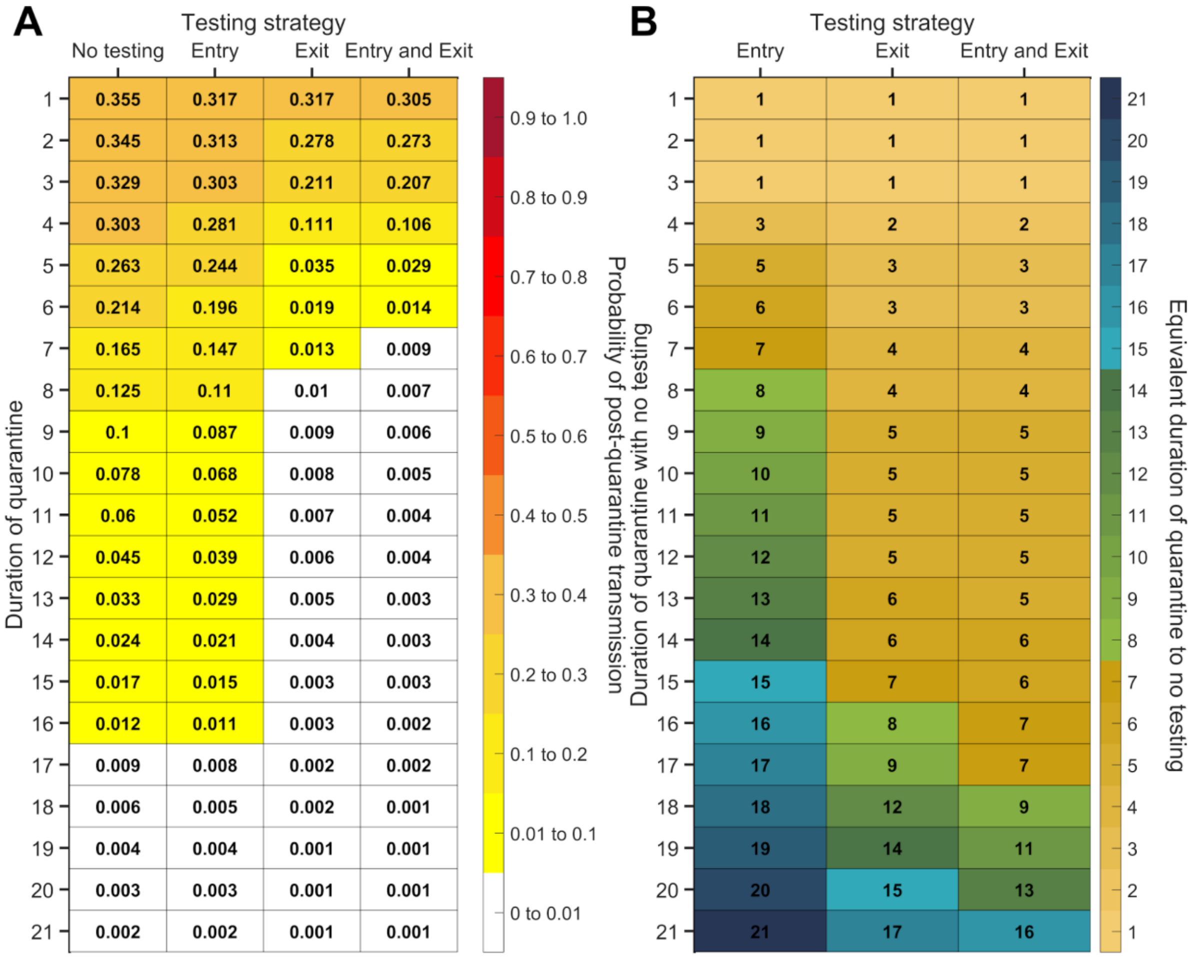


Supplementary Figure 19. **Probability of post-quarantine transmission and equivalent quarantine duration with testing for traced contacts, specifying a latent period of 3.9 days.** For durations of quarantine from 1–21 days, when an individual enters quarantine through contact tracing, with an incubation period of 8.29 days, with 30.8% of infections being asymptomatic, and perfect self-isolation of symptomatic infections, (A) the probability of post-quarantine transmission (probability of one or more post-quarantine infections) with no testing, when tested upon entry to quarantine, when tested on exit from quarantine, and when tested on entry and exit from quarantine, and (B) the durations of quarantine with testing on entry, testing on exit, and testing on entry and exit that perform just as well or better than a quarantine with no testing. Cells that share a background color in common indicate equivalent durations of quarantine associated with each of the testing strategies. Because of the time required to obtain test results, sampling for the test on exit is assumed to occur the day before the quarantine is complete.

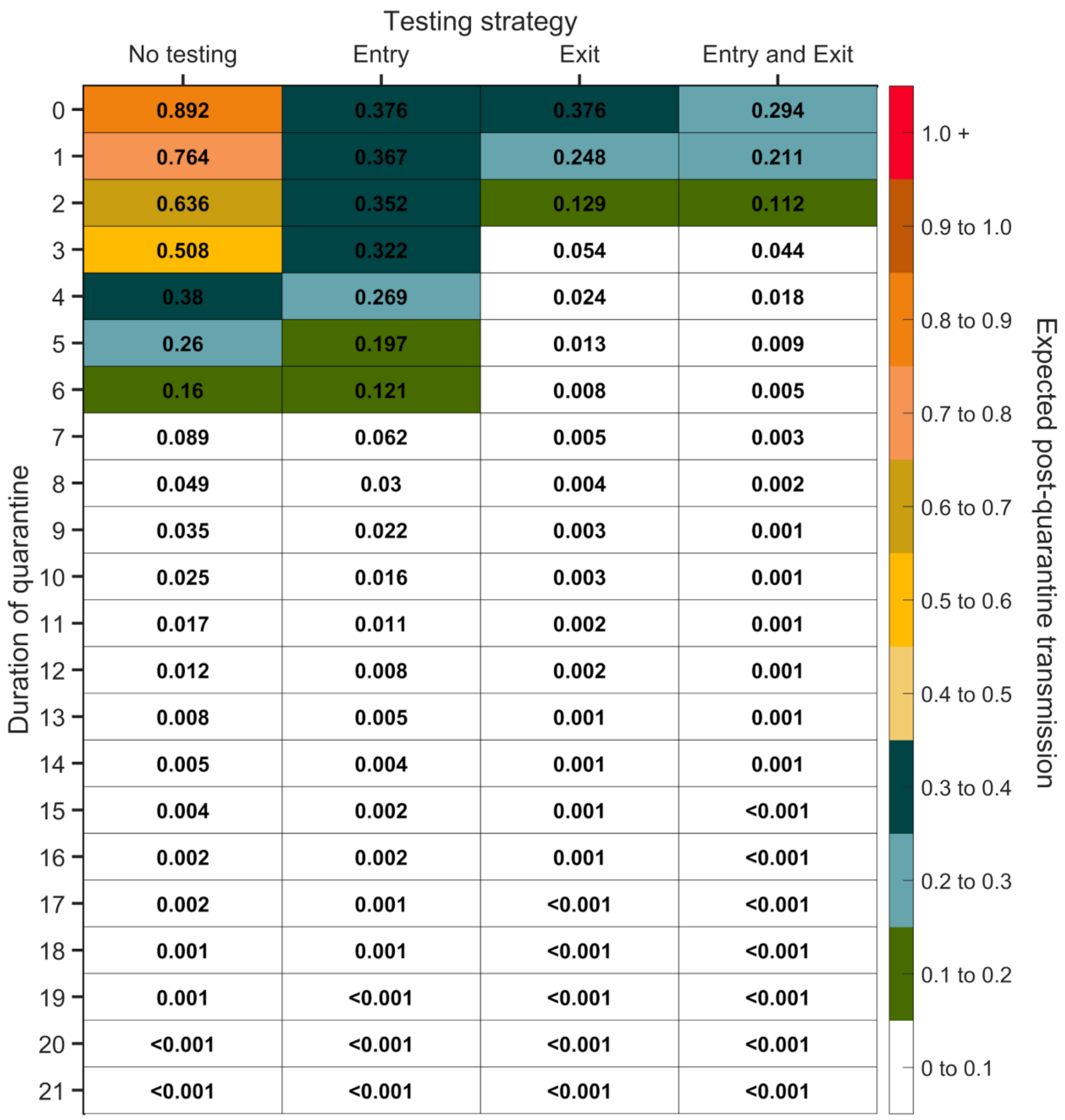


Supplementary Figure 20. **Expected post-quarantine infections for travel quarantine, when there is no delay in obtaining test results.** Expected post-quarantine infections for durations of quarantine of 0–21 days, with an incubation period of 8.29 days, a latent period of 2.9 days, 30.8% of infections being asymptomatic, perfect self-isolation of symptomatic infections when symptomatic, uniform entry within the incubation period by symptomatic cases, and uniform entry across the disease time course for asymptomatic cases, with no testing, testing on entry, testing on exit, and testing on entry and exit. Testing on exit is assumed to occur on the last day of quarantine (i.e. there is negligible delay in obtaining the test result).

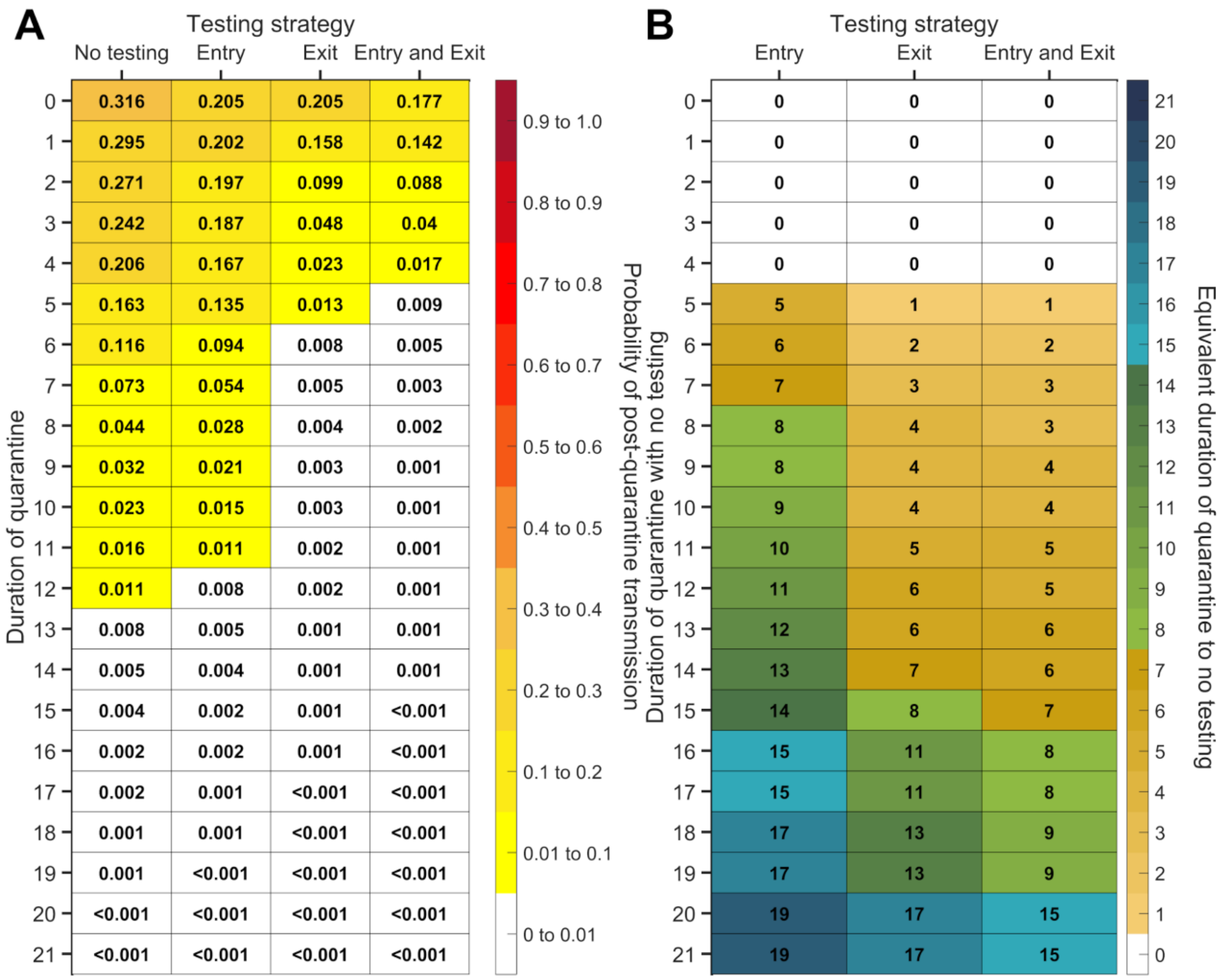


Supplementary Figure 21. **Probability of post-quarantine transmission and equivalent quarantine duration with testing for travel quarantine, when there is no delay in obtaining test results.** For durations of quarantine from 0–21 days, when a symptomatic individual enters quarantine uniformly within the incubation period and asymptomatic individuals enter uniformly across the disease time course, with an incubation period of 8.29 days, a latent period of 2.9 days, with 30.8% of infections being asymptomatic, and perfect self-isolation of symptomatic infections, (A) the probability of post-quarantine transmission (probability of one or more post-quarantine infections) with no testing, when tested upon entry to quarantine, when tested on exit from quarantine, and when tested on entry and exit from quarantine, and (B) the durations of quarantine with testing on entry, testing on exit, and testing on entry and exit that perform just as well or better than a quarantine with no testing. Cells that share a background color in common indicate equivalent durations of quarantine associated with each of the testing strategies. Testing on exit is assumed to occur on the last day of quarantine (i.e. there is a negligible delay in obtaining the test results).

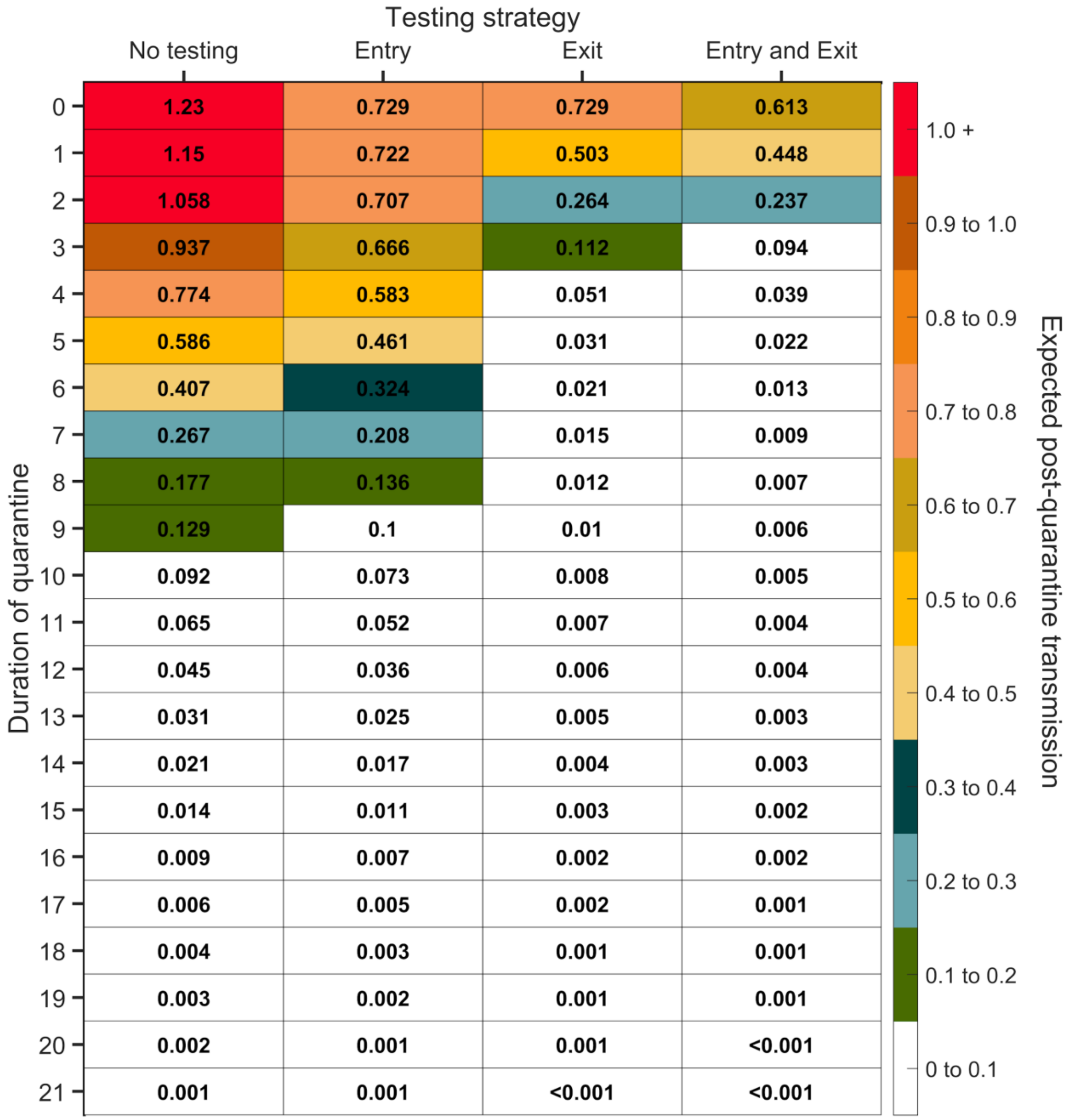


Supplementary Figure 22. **Expected post-quarantine infections for traced contacts, when there is no delay in obtaining test results.** Expected post-quarantine infections for durations of quarantine of 0–21 days, with an incubation period of 8.29 days, a latent period of 2.9 days, 30.8% of infections being asymptomatic, perfect self-isolation of symptomatic infections when symptomatic, and entry through contact tracing, with no testing, testing on entry, testing on exit, and testing on entry and exit. Testing on exit is assumed to occur on the last day of quarantine (i.e. there is negligible delay in obtaining the test result).

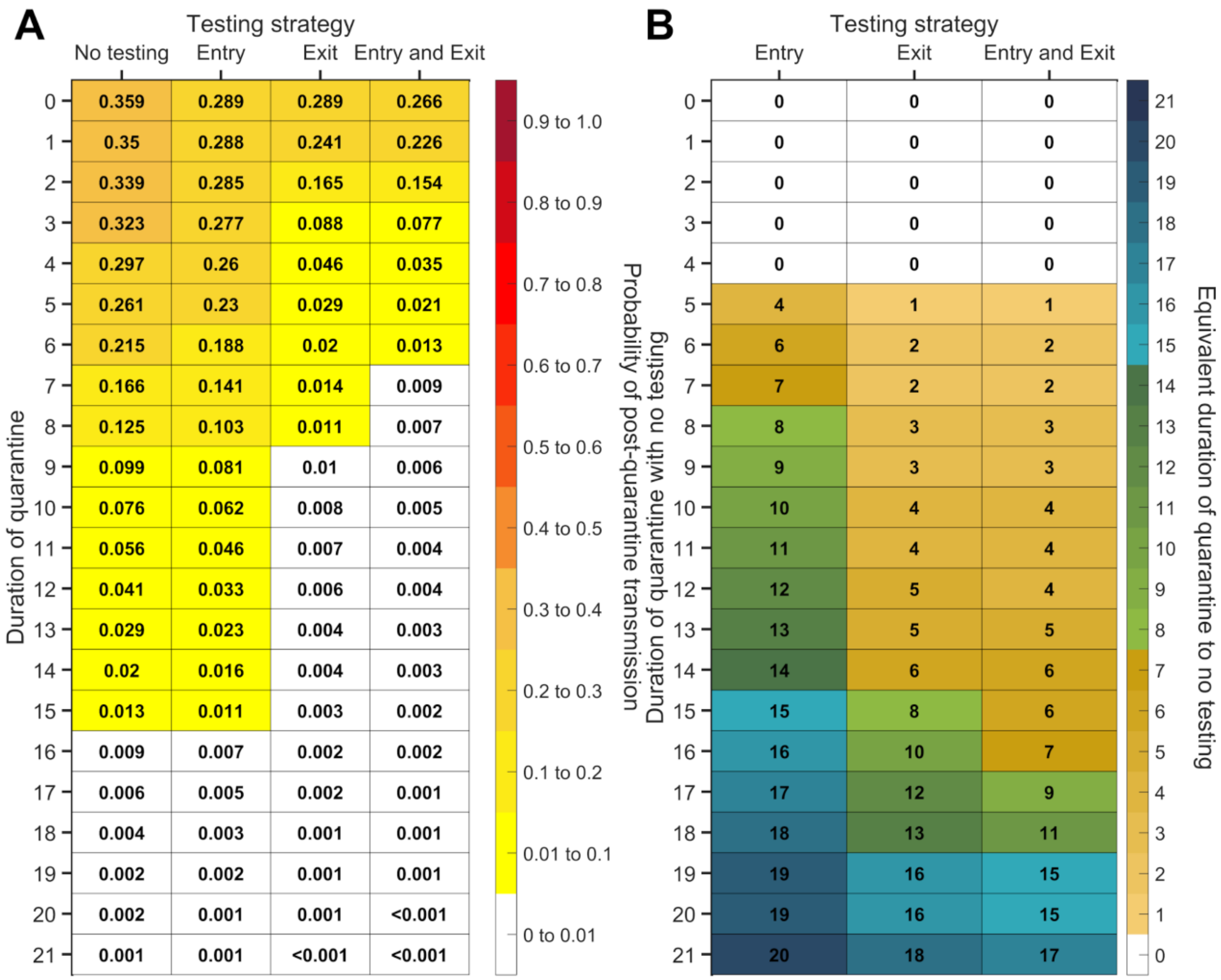


Supplementary Figure 23. **Probability of post-quarantine transmission and equivalent quarantine duration with testing for traced contacts, when there is no delay in obtaining test results.** For durations of quarantine from 0–21 days, when an individual enters quarantine through contact tracing, with an incubation period of 8.29 days, a latent period of 2.9 days, with 30.8% of infections being asymptomatic, and perfect self-isolation of symptomatic infections, (A) the probability of post-quarantine transmission (probability of one or more post-quarantine infections) with no testing, when tested upon entry to quarantine, when tested on exit from quarantine, and when tested on entry and exit from quarantine, and (B) the durations of quarantine with testing on entry, testing on exit, and testing on entry and exit that perform just as well or better than a quarantine with no testing. Cells that share a background color in common indicate equivalent durations of quarantine associated with each of the testing strategies. Testing on exit is assumed to occur on the last day of quarantine (i.e. there is a negligible delay in obtaining the test result).

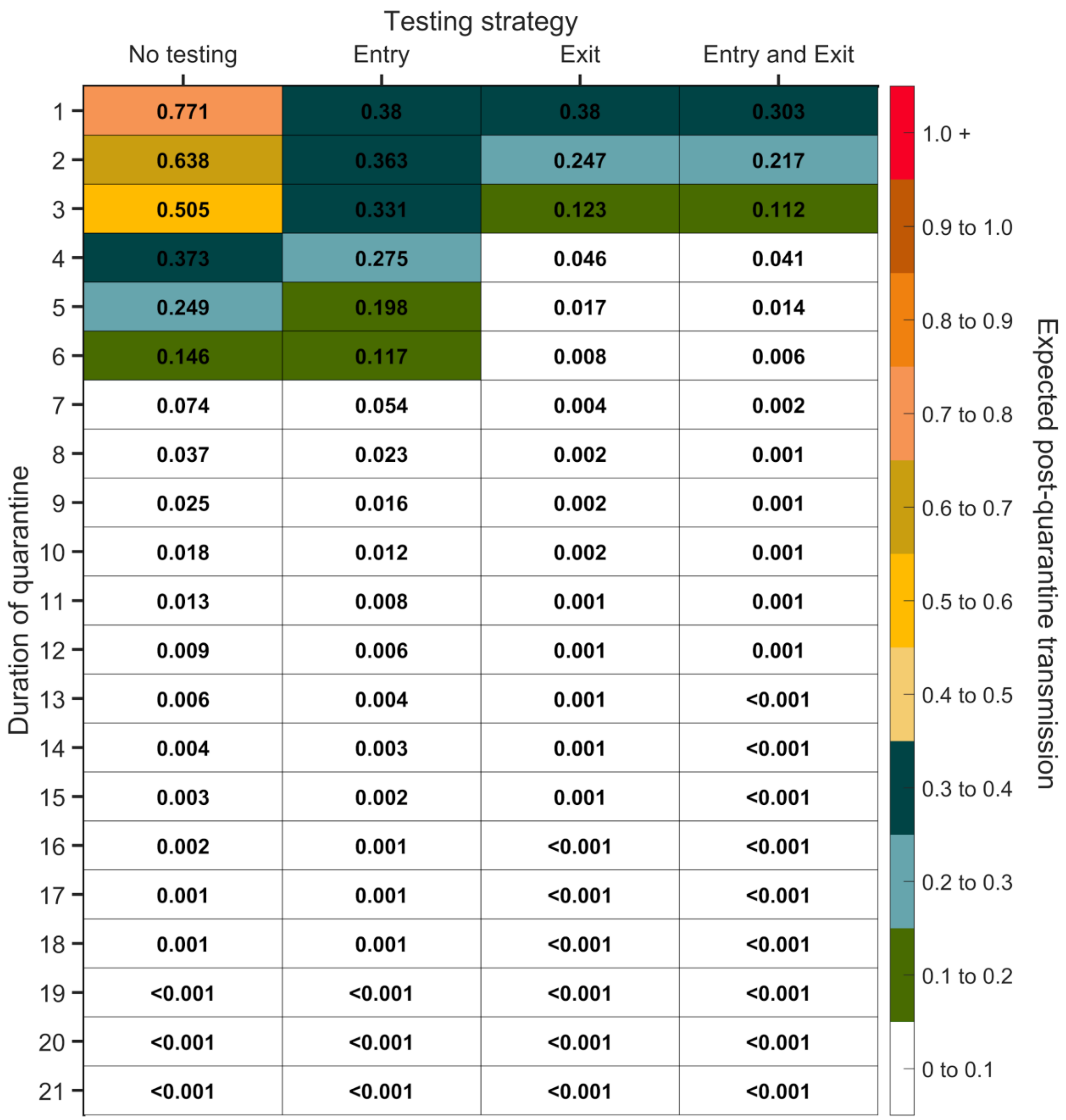


Supplementary Figure 24. **Expected post-quarantine infections for travel quarantine, specifying 22.6%of infections as asymptomatic.** Expected post-quarantine infections for durations of quarantine of 1–21 days, with an incubation period of 8.29 days, a latent period of 2.9 days, perfect self-isolation of symptomatic infections when symptomatic, uniform entry within the incubation period by symptomatic cases, and uniform entry across the disease time course for asymptomatic cases, with no testing, testing on entry, testing on exit, and testing on entry and exit. Because of the time required to obtain test results, sampling for the test on exit was assumed to occur the day before the quarantine was completed.

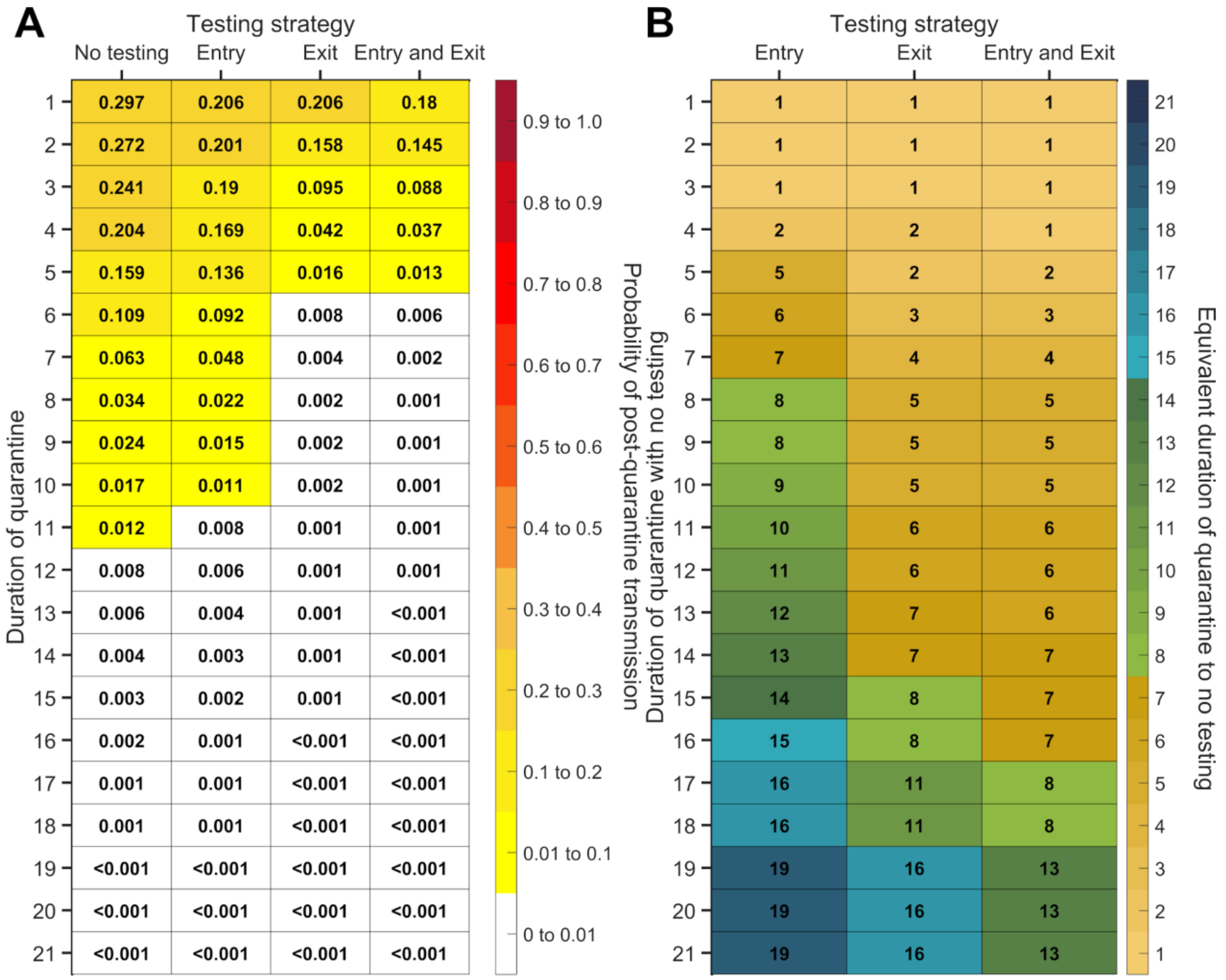


Supplementary Figure 25. **Probability of post-quarantine transmission and equivalent quarantine duration with testing for travel quarantine, specifying 22.6% of infections as asymptomatic.** For durations of quarantine from 1–21 days, when a symptomatic individual enters quarantine uniformly within the incubation period and asymptomatic individuals enter uniformly across the disease time course, with an incubation period of 8.29 days, a latent period of 2.9 days, and perfect self-isolation of symptomatic infections, (A) the probability of post-quarantine transmission (probability of one or more post-quarantine infections) with no testing, when tested upon entry to quarantine, when tested on exit from quarantine, and when tested on entry and exit from quarantine, and (B) the durations of quarantine with testing on entry, testing on exit, and testing on entry and exit that perform just as well or better than a quarantine with no testing. Cells that share a background color in common indicate equivalent durations of quarantine associated with each of the testing strategies. Because of the time required to obtain test results, sampling for the test on exit is assumed to occur the day before the quarantine is complete.

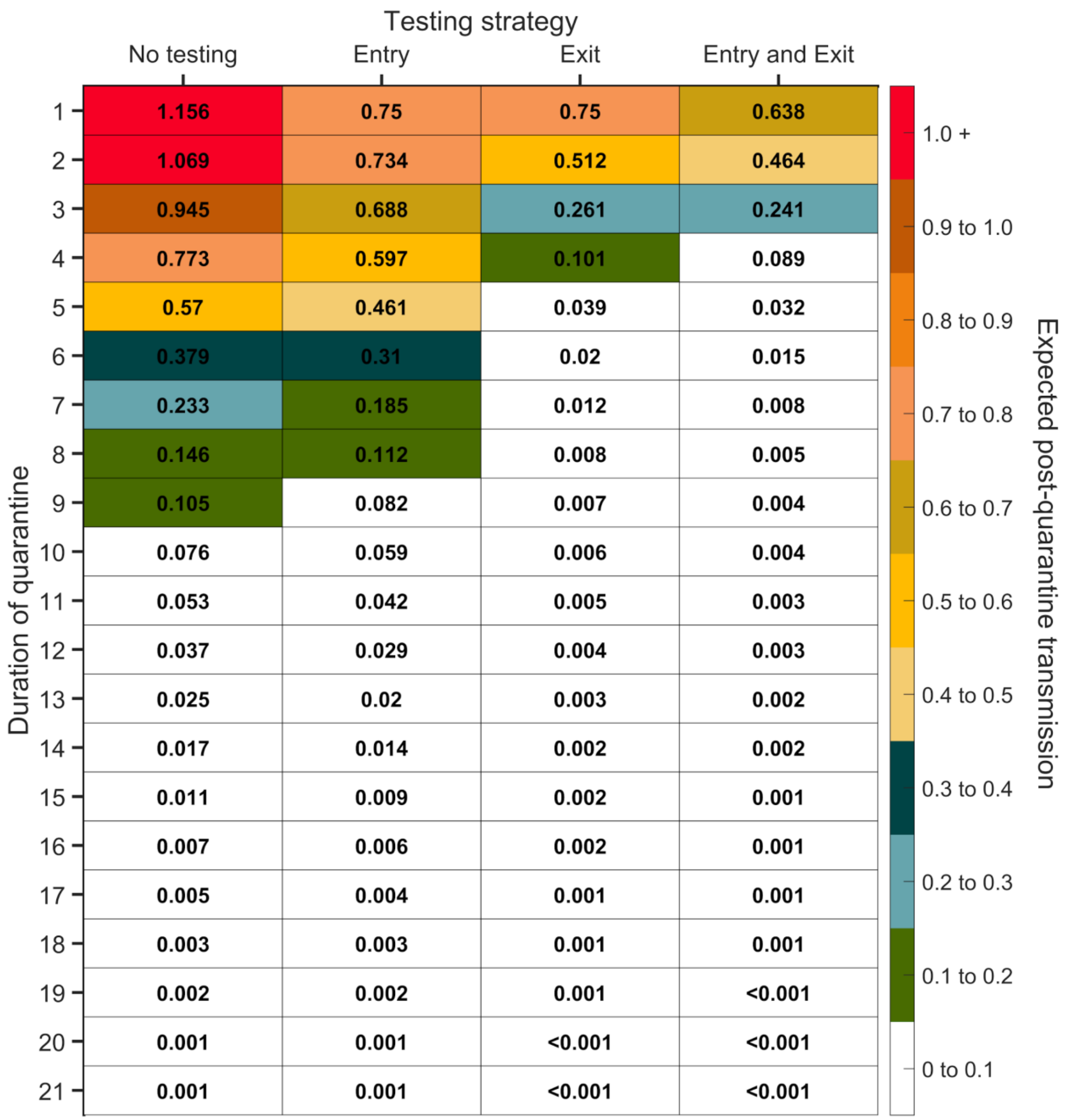


Supplementary Figure 26. **Expected post-quarantine infections for traced contacts, specifying 22.6% of infections as asymptomatic.** Expected post-quarantine infections for durations of quarantine of 1–21 days, with an incubation period of 8.29 days, a latent period of 2.9 days, perfect self-isolation of symptomatic infections when symptomatic, and entry through contact tracing, with no testing, testing on entry, testing on exit, and testing on entry and exit. Because of the time required to obtain test results, sampling for the test on exit was assumed to occur the day before the quarantine was completed.

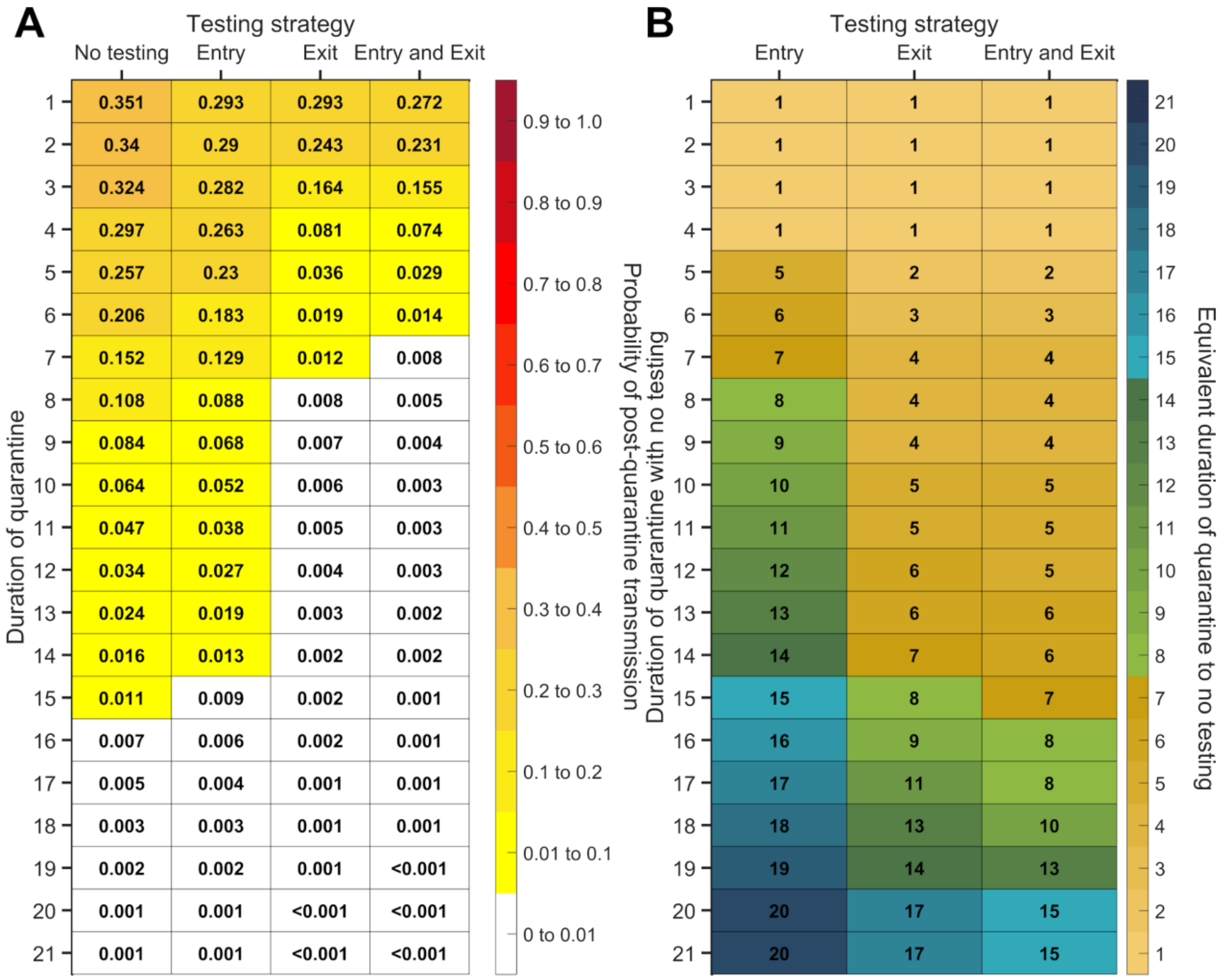


Supplementary Figure 27. **Probability of post-quarantine transmission and equivalent quarantine duration with testing for traced contacts, specifying 22.6% of infections as asymptomatic.** For durations of quarantine from 1–21 days, when an individual enters quarantine through contact tracing, with an incubation period of 8.29 days, a latent period of 2.9 days, and perfect self-isolation of symptomatic infections, (A) the probability of post-quarantine transmission (probability of one or more post-quarantine infections) with no testing, when tested upon entry to quarantine, when tested on exit from quarantine, and when tested on entry and exit from quarantine, and (B) the durations of quarantine with testing on entry, testing on exit, and testing on entry and exit that perform just as well or better than a quarantine with no testing. Cells that share a background color in common indicate equivalent durations of quarantine associated with each of the testing strategies. Because of the time required to obtain test results, sampling for the test on exit is assumed to occur the day before the quarantine is complete.

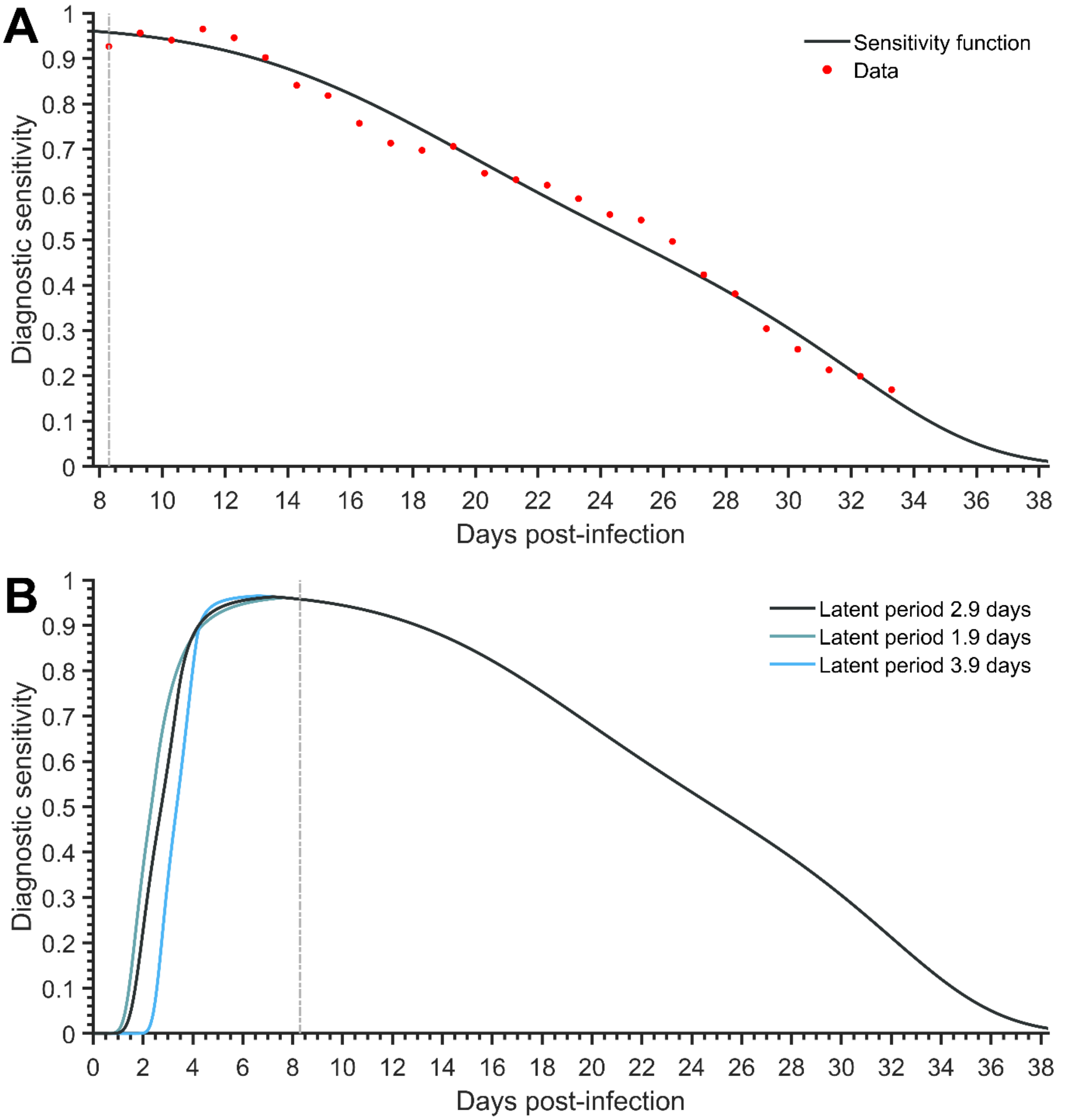


Supplementary Figure 28. **Temporal diagnostic sensitivity of the RT-PCR test.** For an incubation period of 8.29 days, the diagnostic sensitivity of the RT-PCR test over the time course of disease (A) determined using a logistic regression model (black line) fit to the empirical data of SARS CoV-2 test results from Miller et al [6](red dots) through minimization of least squares and AIC model selection, and (B) specifying latent periods of 2.9 days (black), 1.9 days (green), and 3.9 days (blue).

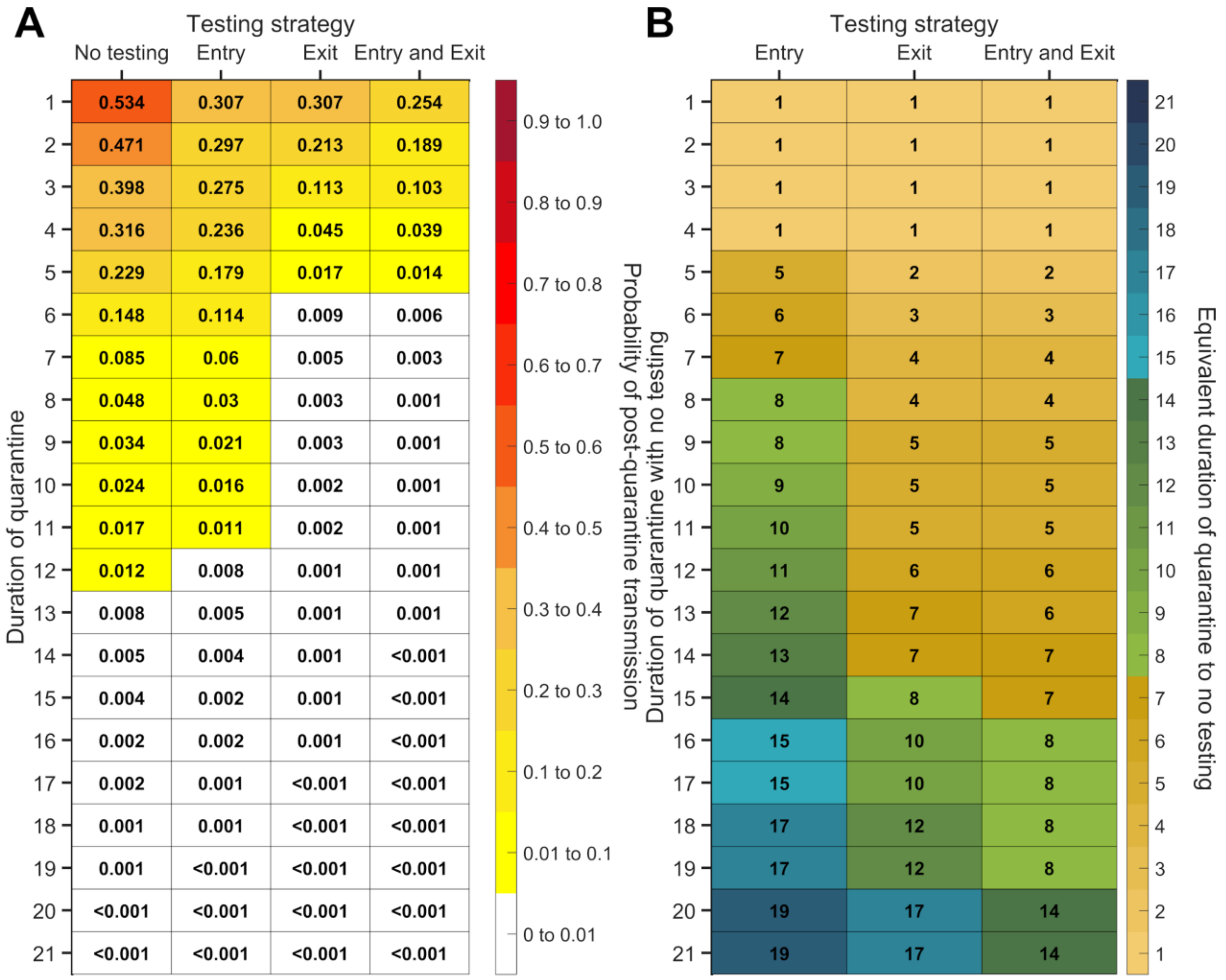


Supplementary Figure 29. **Probability of post-quarantine transmission and equivalent quarantine duration with testing for travel quarantine, assuming infections are Poisson distributed.** For durations of quarantine from 1–21 days, when a symptomatic individual enters quarantine uniformly within the incubation period and asymptomatic individuals enter uniformly across the disease time course, with an incubation period of 8.29 days, a latent period of 2.9 days, with 30.8% of infections being asymptomatic, and perfect self-isolation of symptomatic infections, (A) the probability of post-quarantine transmission (probability of one or more post-quarantine infections), assuming infections are Poisson distributed, with no testing, when tested upon entry to quarantine, when tested on exit from quarantine, and when tested on entry and exit from quarantine, and (B) the durations of quarantine with testing on entry, testing on exit, and testing on entry and exit that perform just as well or better than a quarantine with no testing. Cells that share a background color in common indicate equivalent durations of quarantine associated with each of the testing strategies. Because of the time required to obtain test results, sampling for the test on exit is assumed to occur the day before the quarantine is complete.

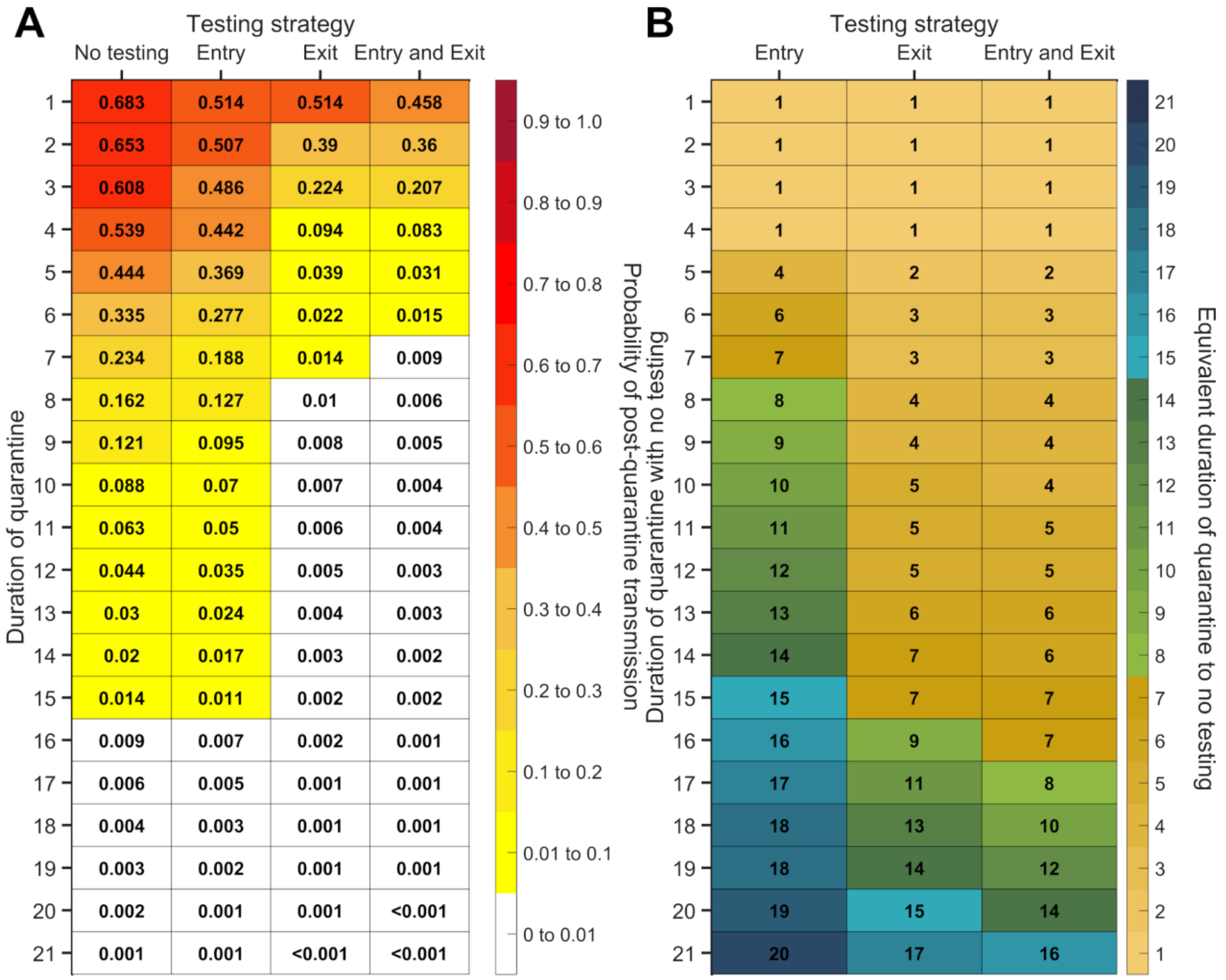


Supplementary Figure 30. **Probability of post-quarantine transmission and equivalent quarantine duration with testing for traced contacts, assuming infections are Poisson distributed.** For durations of quarantine from 1–21 days, when an individual enters quarantine through contact tracing, with an incubation period of 8.29 days, a latent period of 2.9 days, with 30.8% of infections being asymptomatic, and perfect self-isolation of symptomatic infections, (A) the probability of post-quarantine transmission (probability of one or more post-quarantine infections), assuming infections are Poisson distributed, with no testing, when tested upon entry to quarantine, when tested on exit from quarantine, and when tested on entry and exit from quarantine, and (B) the durations of quarantine with testing on entry, testing on exit, and testing on entry and exit that perform just as well or better than a quarantine with no testing. Cells that share a background color in common indicate equivalent durations of quarantine associated with each of the testing strategies. Because of the time required to obtain test results, sampling for the test on exit is assumed to occur the day before the quarantine is complete.

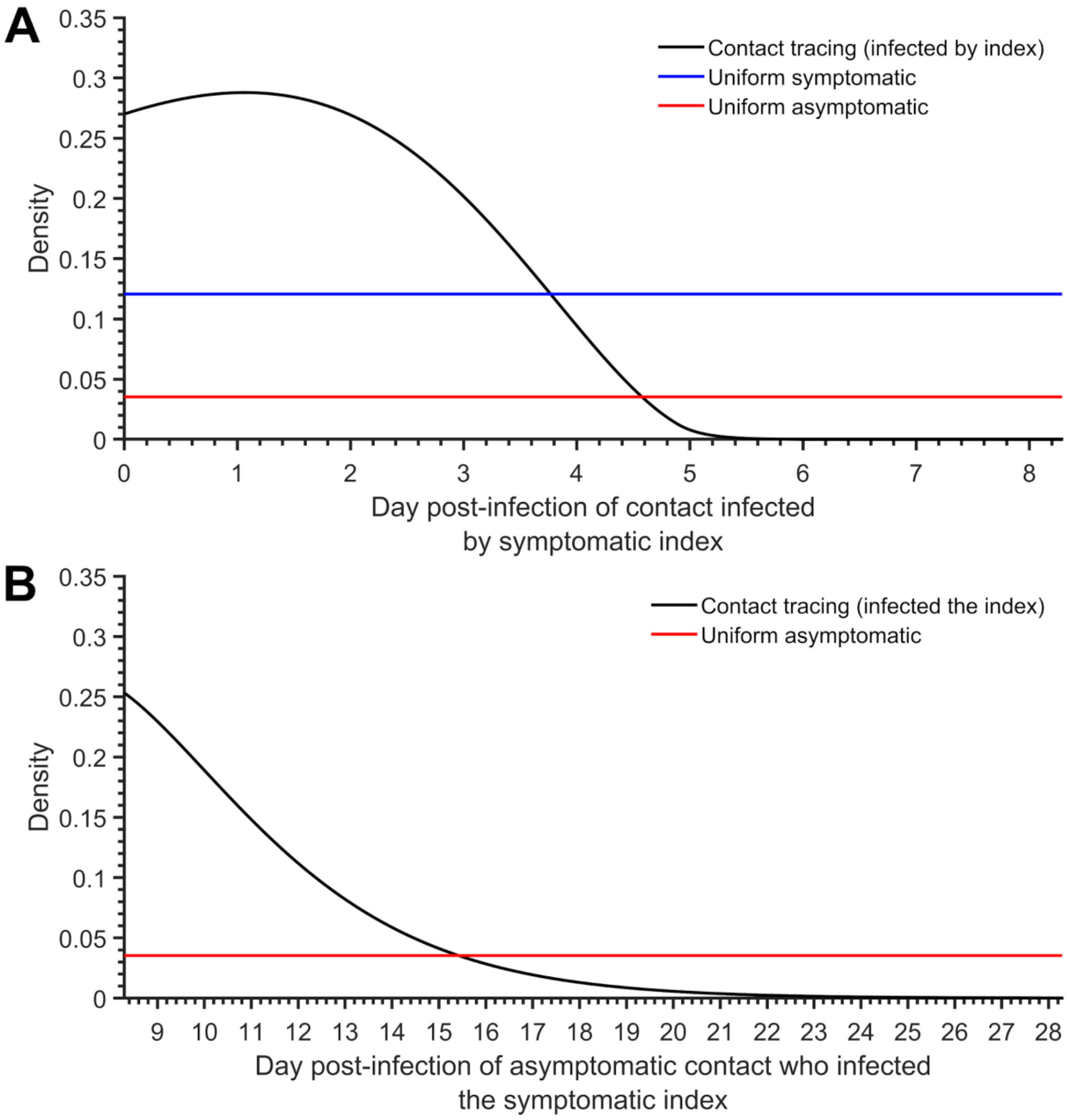


Supplementary Figure 31. **Probability densities of the time post-infection that an individual enters quarantine.** Probability density functions for when during the disease time course cases enter quarantine, including (A) the day of disease time course in which a contact infected by an index case enters quarantine (black) compared to the uniform entry into quarantine of a case to exhibit symptoms (blue) and an asymptomatic case (red), and (B) the day of disease time course in which the asymptomatic contact that infected the index case enters quarantine (black) compared to the uniform entry into quarantine of an asymptomatic case (red).

## Supplementary References

1. Li, Y. *et al.* Asymptomatic and Symptomatic Patients With Non-severe Coronavirus Disease (COVID-19) Have Similar Clinical Features and Virological Courses: A Retrospective Single Center Study. *Front. Microbiol.* **11**, 1570 (2020).
2. Lee, S. *et al.* Clinical Course and Molecular Viral Shedding Among Asymptomatic and Symptomatic Patients With SARS-CoV-2 Infection in a Community Treatment Center in the Republic of Korea. *JAMA Intern. Med.* **180**, 1447–1452 (2020).
3. Zhang, Y., Li, Y., Wang, L., Li, M. & Zhou, X. Evaluating Transmission Heterogeneity and Super-Spreading Event of COVID-19 in a Metropolis of China. *Int. J. Environ. Res. Public Health* **17**, (2020).
4. He, X. *et al.* Temporal dynamics in viral shedding and transmissibility of COVID-19. *Nat. Med.* **26**, 672–675 (2020).
5. Meeker, W. Q. & Escobar, L. A. Teaching about Approximate Confidence Regions Based on Maximum Likelihood Estimation. *The American Statistician* **49**, 48 (1995).
6. Miller, T. E. *et al.* Clinical sensitivity and interpretation of PCR and serological COVID-19 diagnostics for patients presenting to the hospital. *FASEB J.* **34**, 13877–13884 (2020).
7. Moghadas, S. M. *et al.* Projecting hospital utilization during the COVID-19 outbreaks in the United States. *Proc. Natl. Acad. Sci. U. S. A.* **117**, 9122–9126 (2020).
8. Qin, J. *et al.* Estimation of incubation period distribution of COVID-19 using disease onset forward time: A novel cross-sectional and forward follow-up study. *Sci Adv* **6**, eabc1202 (2020).
9. Xiao, A. T. *et al.* Dynamic profile of RT-PCR findings from 301 COVID-19 patients in Wuhan, China: A descriptive study. *J. Clin. Virol.* **127**, 104346 (2020).

10. CDC. Duration of Isolation and Precautions for Adults with COVID-19. https://www.cdc.gov/coronavirus/2019-ncov/hcp/duration-isolation.html (2020).
11. Cevik M, Tate M, Lloyd O, Maraolo AE, Schafers J, Ho A. SARS-CoV-2, SARS-CoV, and MERS-CoV viral load dynamics, duration of viral shedding, and infectiousness: a systematic review and meta-analysis. *The Lancet Microbe* (2020) doi:10.1016/S2666-5247(20)30172-5.
12. Nishiura, H. *et al.* Estimation of the asymptomatic ratio of novel coronavirus infections (COVID-19). *Int. J. Infect. Dis.* **94**, 154–155 (2020).
13. Buitrago-Garcia, D. C. *et al.* Asymptomatic SARS-CoV-2 infections: a living systematic review and meta-analysis. *Preprint at https://www.medrxiv.org/content/10.1101/2020.04.25.20079103v3.abstract* (2020) doi:10.1101/2020.04.25.20079103.
14. Wang, Y. *et al.* Characterization of an Asymptomatic Cohort of Severe Acute Respiratory Syndrome Coronavirus 2 (SARS-CoV-2) Infected Individuals Outside of Wuhan, China. *Clinical Infectious Diseases* **71**, 2132–2138 (2020).
15. Wang, X. *et al.* Impact of Social Distancing Measures on Coronavirus Disease Healthcare Demand, Central Texas, USA. *Emerg. Infect. Dis.* **26**, 2361–2369 (2020).
