# Supplementary material for: Optimal COVID-19 quarantine and testing strategies
Source: Nat Commun. 2021 Jan 7;12:356. doi: 10.1038/s41467-020-20742-8 (PMC7788536; doi:10.1038/s41467-020-20742-8)
Supplement: Supplementary file 2 — Reporting Summary [file 41467_2020_20742_MOESM2_ESM.pdf]

Corresponding author(s): Dr. Jeffrey P. Townsend

Last updated by author(s): December 14, 2020

Reporting Summary

Nature Research wishes to improve the reproducibility of the work that we publish. This form provides structure for consistency and transparency in reporting. For further information on Nature Research policies, see our Editorial Policies and the Editorial Policy Checklist.

Statistics

For all statistical analyses, confirm that the following items are present in the figure legend, table legend, main text, or Methods section.

n/a	Confirmed	
☐	☒	The exact sample size (n) for each experimental group/condition, given as a discrete number and unit of measurement
☒	☐	A statement on whether measurements were taken from distinct samples or whether the same sample was measured repeatedly
☒	☐	The statistical test(s) used AND whether they are one- or two-sided<br>*Only common tests should be described solely by name; describe more complex techniques in the Methods section.*
☒	☐	A description of all covariates tested
☒	☐	A description of any assumptions or corrections, such as tests of normality and adjustment for multiple comparisons
☒	☐	A full description of the statistical parameters including central tendency (e.g. means) or other basic estimates (e.g. regression coefficient) AND variation (e.g. standard deviation) or associated estimates of uncertainty (e.g. confidence intervals)
☒	☐	For null hypothesis testing, the test statistic (e.g. F, t, r) with confidence intervals, effect sizes, degrees of freedom and P value noted<br>*Give P values as exact values whenever suitable.*
☒	☐	For Bayesian analysis, information on the choice of priors and Markov chain Monte Carlo settings
☒	☐	For hierarchical and complex designs, identification of the appropriate level for tests and full reporting of outcomes
☒	☐	Estimates of effect sizes (e.g. Cohen's d, Pearson's r), indicating how they were calculated

Our web collection on statistics for biologists contains articles on many of the points above.

Software and code

Policy information about availability of computer code

Data collection	For some of the theory, the digitization of curves from the referenced publications in the manuscript was done using WebPlotDigitizer (Version 4.3) (https://automeris.io/WebPlotDigitizer/). For other data in the manuscript, no software was used in the collection of data.
Data analysis	MATLAB R2018b was used to generate the figures for the empirically collected data, and conduct the numerical analysis of the theory component of the manuscript. he computational code for the analysis was implemented in MATLAB, and it is available at github.com/WellsRC/Optimizing-COVID19-Quarantine-and-Testing-Strategies.

For manuscripts utilizing custom algorithms or software that are central to the research but not yet described in published literature, software must be made available to editors and reviewers. We strongly encourage code deposition in a community repository (e.g. GitHub). See the Nature Research guidelines for submitting code & software for further information.

Data

Policy information about availability of data

All manuscripts must include a data availability statement. This statement should provide the following information, where applicable:

- Accession codes, unique identifiers, or web links for publicly available datasets
- A list of figures that have associated raw data
- A description of any restrictions on data availability

The number of positive tests and tests conducted at the two regions quarantining the crew members heading offshore are presented in Fig. 2, with other data used in the analysis referenced in Supplementary Table 1 and in the Methods.

Field-specific reporting

Please select the one below that is the best fit for your research. If you are not sure, read the appropriate sections before making your selection.

☒ Life sciences ☐ Behavioural & social sciences ☐ Ecological, evolutionary & environmental sciences

For a reference copy of the document with all sections, see nature.com/documents/nr-reporting-summary-flat.pdf

Life sciences study design

All studies must disclose on these points even when the disclosure is negative.

Sample size	No sample size calculation was conducted. The sample sizes were based on the available data of groups of crew members heading off shore between April 11, 2020 and August 26, 2020. For the assessment of the testing strategies, the minimum sample size was 110 for the one region. For the follow-up testing, during the early phase there were 37 individuals tested and in a later phase 155.
Data exclusions	Between April 11, 2020 and August 26, 2020, there were 4,040 SARS CoV-2 RT-PCR tests conducted among employees of an oil and gas company coming from two regions (stratified by lab location). A third region that was monitored is not included in our data set, as there was low population prevalence entering quarantine and there were no positive tests.
Replication	Since the data obtained from the oil and gas company is dependent on the stage of the epidemic and the background prevalence of COVID-19 in the population entering quarantine it would be difficult to reproduce quantitatively.
Randomization	Randomization was not relevant to our study, as the companies strategy evolved over time based on the information they were receiving.
Blinding	Blinding was not relevant (or possible) as crew members were screened for infection with testing prior to heading to the off-shore site. Prior to entering quarantine, individuals had to pass a screening survey to further mitigate any risk.

Reporting for specific materials, systems and methods

We require information from authors about some types of materials, experimental systems and methods used in many studies. Here, indicate whether each material, system or method listed is relevant to your study. If you are not sure if a list item applies to your research, read the appropriate section before selecting a response.

Materials & experimental systems

n/a	Involved in the study	
☒	☐	Antibodies
☒	☐	Eukaryotic cell lines
☒	☐	Palaeontology and archaeology
☒	☐	Animals and other organisms
☐	☒	Human research participants
☒	☐	Clinical data
☒	☐	Dual use research of concern

Methods

n/a	Involved in the study	
☒	☐	ChIP-seq
☒	☐	Flow cytometry
☒	☐	MRI-based neuroimaging

Human research participants

Policy information about studies involving human research participants

Population characteristics	Between April 11, 2020 and August 26, 2020, there were 4,040 SARS CoV-2 RT-PCR tests conducted among employees of an oil and gas company coming from two regions (stratified by lab location). A third region that was monitored is not included in our data set, as there was low population prevalence entering quarantine and there were no positive tests.<br><br>The crew members were 97% male and had an average age of 34 years, ranging from 24 to 64 years of age.
Recruitment	Recruitment was based on crew members entering quarantine and being tested prior to heading to the off-shore site. During the early stages of the epidemic, both regions used a three-day quarantine with testing on entry. On August 13, employees from region A quarantined at home for seven days, with testing occurring on both entry and exit. While employees were at home, they were asked to practice social distancing in public. Starting on June 25, employees from region B were quarantined in a hotel for five days prior to their departure off-shore and tested on both entry and exit. The requirements of an employee to enter quarantine were (1) passing the components of a screening form used to filter out symptomatic cases and those potentially exposed and (2) temperature screenings.<br><br>With the screening process filtering out those potentially exposed recently but not yet symptomatic, the test positivity rate on entry is expected to increase compared to what has been observed, as these screened individuals would be in the incubation period where diagnostic sensitivity can be lower.

	The timing of the testing relative to the epidemic trajectory will also influence the proportion of cases entering in the incubation period. In the early stages of the epidemic, most cases are concentrated in the incubation period (or early stages of disease). This early entry would decrease the test positivity rate on entry. As the epidemic trend starts to decline, individuals entering quarantine will more likely be in the later stages of disease, which can influence the positivity rate on the entry and exit test.
Ethics oversight	For the test ordered by XstremeMD (offshore group that collected the tests), each crew member signed a HIPPA release. The Human Participants Review Sub-Committee, York University's Ethics Review Board (Certificate Number: 2020-323).

Note that full information on the approval of the study protocol must also be provided in the manuscript.
